# Unexpected Remyelination in the Absence of Matrix Metalloproteinase 7

**DOI:** 10.1002/glia.70005

**Published:** 2025-03-10

**Authors:** Rianne P. Gorter, Andrea J. Arreguin, Wendy Oost, Jenny C. de Jonge, Harm H. Kampinga, Sandra Amor, Holly Colognato, Wia Baron

**Affiliations:** ^1^ Department of Biomedical Sciences University of Groningen, University Medical Center Groningen Groningen the Netherlands; ^2^ Queen Mary University of London MS Center Noord Nederland Groningen the Netherlands; ^3^ Department of Pharmacological Sciences Stony Brook University Stony Brook New York USA; ^4^ Department of Pathology Amsterdam UMC‐Location VUmc Amsterdam the Netherlands; ^5^ Blizard Institute, Barts and the London School of Medicine and Dentistry Department of Neuroscience and Trauma Queen Mary University of London London UK; ^6^ Institute of Anatomy Rostock University Medical Center Rostock Germany

**Keywords:** cuprizone, demyelination, extracellular matrix, fibronectin, hyaluronan, matrix metalloproteinase 7, multiple sclerosis, remyelination

## Abstract

In multiple sclerosis (MS), an influx of immune cells into the central nervous system leads to focal demyelinating lesions in the brain, optic nerve, and spinal cord. As MS progresses, remyelination increasingly fails, leaving neuronal axons vulnerable to degeneration and resulting in permanent neurological disability. In chronic MS lesions, the aberrant accumulation of extracellular matrix (ECM) molecules, including fibronectin and hyaluronan, impairs oligodendrocyte progenitor cell differentiation, contributing to remyelination failure. Removing inhibitory ECM is therefore a therapeutic target to stimulate remyelination in MS. Intriguingly, the expression of the fibronectin‐degrading enzyme matrix metalloproteinase 7 (MMP7) is decreased in chronic MS lesions compared to control white matter. Therefore, we examined the role of MMP7 upon cuprizone‐induced demyelination, hypothesizing that the lack of MMP7 would lead to impaired breakdown of its ECM substrates, including fibronectin, and diminished remyelination. Unexpectedly, remyelination proceeded efficiently in the absence of MMP7. In the remyelination phase, the lack of MMP7 did not lead to the accumulation of fibronectin or of laminin, another MMP7 substrate. Moreover, in the setting of chronic demyelination, levels of fibronectin were actually lower in MMP7^−/−^ mice, while levels of hyaluronan, which is not a known MMP7 substrate, were also lower. Overall, these results indicate that MMP7 is not essential for remyelination in the cuprizone model and point to an unexpected complexity in how MMP7 deficiency influences fibronectin and hyaluronan levels in chronic demyelination.

## Main Points

- MMP7 is not essential for demyelination and remyelination in the cuprizone model.
- MMP7^‐/‐^ mice fibronectin aggregates do not accumulate in the remyelination phase.
- MMP7^‐/‐^ mice have lower levels of fibronectin and hyaluronan after chronic demyelination.

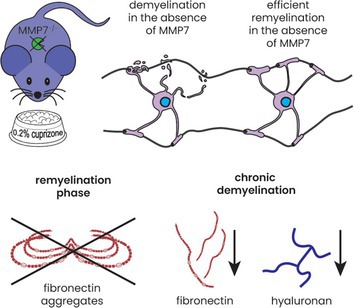

## 1 Introduction

Multiple sclerosis (MS) is a chronic demyelinating disease of the central nervous system (CNS), characterized by diffuse abnormalities in the gray and white matter, as well as focal lesions with varying degrees of inflammation, demyelination, glial scar formation, and axonal loss (Trapp and Nave 2008; Lassmann 2018). The resulting neurological symptoms may initially appear and disappear in a relapsing–remitting pattern (Kuhlmann et al. 2023). This recovery is at least in part due to remyelination: the process in which (newly formed) oligodendrocytes rewrap denuded neuronal axons in myelin, restoring saltatory conduction and preventing degeneration of the neuronal‐axonal unit (Franklin and Simons 2022). Unfortunately, over time, the continual damage to the CNS outweighs intrinsic repair mechanisms, leading to the progressive accumulation of neurological disability (Kuhlmann et al. 2023). Stimulating remyelination would be one avenue to delay, or even halt, disease progression in MS.

Although oligodendrocyte progenitor cells (OPCs) are found in the majority of chronically demyelinated MS lesions (Lucchinetti et al. 1999; Chang et al. 2002), their presence does not always translate to efficient remyelination (Luchetti et al. 2018). Here, detrimental cues from the microenvironment likely contribute to deficient OPC differentiation (Franklin and Simons 2022). In particular, changes in the composition of the extracellular matrix (ECM), that is, the tissue‐specific network of glycoproteins, proteoglycans, and polysaccharides that surrounds and interacts with cells, affect OPC differentiation and remyelination (Ghorbani and Yong 2021). For example, astrocytes in chronically demyelinated MS lesions produce high molecular weight (HMW) hyaluronan (Back et al. 2005), a polysaccharide that inhibits OPC differentiation when it is degraded into smaller fragments (Sloane et al. 2010). The ECM protein fibronectin also regulates remyelination dynamics (Stoffels et al. 2015; Stoffels, de Jonge, et al. 2013; Sobel and Mitchell 1989; van Horssen et al. 2005). Upon injury in a wide array of tissues, fibroblast‐like cells secrete a provisional fibronectin matrix to which other ECM proteins can bind, thus initiating tissue repair (Stoffels, Zhao, and Baron 2013). Over time, the formation of intermolecular bonds between fibronectin dimers as well as between fibronectin and other ECM molecules (Chung and Erickson 1997; Raitman et al. 2018; Hill et al. 2022) can convert the provisional fibronectin matrix into highly stable fibrils that are insoluble in the detergent deoxycholate (DOC) (Mao and Schwarzbauer 2005). Intriguingly, in models of toxin‐induced demyelination, fibronectin expression is only transiently upregulated by astrocytes in the form of dimers (Stoffels, de Jonge, et al. 2013) or DOC‐soluble multimers (Stoffels, de Jonge, et al. 2013; Espitia Pinzon et al. 2017) and here, the clearance of fibronectin correlates with efficient remyelination. However, in inflammatory models, such as experimental autoimmune encephalitis (EAE) (Stoffels, de Jonge, et al. 2013) and blunt spinal cord injury (Cooper et al. 2018), as well as in chronically demyelinated MS lesions (Stoffels, de Jonge, et al. 2013), DOC‐insoluble fibronectin persists. When these DOC‐insoluble fibronectin multimers, termed *fibronectin aggregates* (Stoffels, de Jonge, et al. 2013), are injected into demyelinated lesions in the rodent brain, remyelination is halted (Stoffels et al. 2015; Qin et al. 2017). Thus, clearing fibronectin aggregates from chronic MS lesions may be a prerequisite for remyelination.

Matrix metalloproteinases (MMPs) are a family of zinc‐dependent proteases that help shape the extracellular environment (Gorter and Baron 2020). MMPs are secreted as proenzymes and require cleavage, often by other MMPs, for their activation (Gorter and Baron 2020). In humans, there are at least 24 different MMPs, which together degrade most components of the ECM (Gorter and Baron 2020). In addition, MMPs cleave many other substrates, including cytokines, receptors, and proteases, leading to their activation or degradation (Gorter and Baron 2020). In MS, one MMP of interest is MMP7, a particularly powerful metalloproteinase that lacks a classical hemopexin‐like C‐terminal domain common to other MMPs (Baragi et al. 1994). This alteration allows MMP7 to cleave a wide array of substrates and renders it less sensitive to inhibition by tissue inhibitors of metalloproteinases (TIMPs) (Baragi et al. 1994). Certain genetic variants in the MMP7 gene are associated with an increased risk of developing MS and a higher disability score within a population of MS patients (Rahimi et al. 2014, 2016). Intriguingly, although (pro)MMP7 expression is upregulated in microglia/macrophages in actively demyelinating MS lesions (Cossins et al. 1997; Anthony et al. 1997; Wang et al. 2018), its expression is severely reduced in chronically demyelinated lesions (Wang et al. 2018). MMP7 efficiently cleaves many ECM proteins, including fibronectin, and is the only MMP known to degrade fibronectin aggregates (Wang et al. 2018; Bredow et al. 1995). Therefore, we hypothesized that lack of MMP7 in these chronic MS lesions contributes to the accumulation of fibronectin, leading to the formation of remyelination‐inhibiting fibronectin aggregates. Previous research shows that deletion of MMP7 prevents the onset of EAE, likely by reducing monocyte and neutrophil infiltration across the blood–brain barrier (Buhler et al. 2009; Li et al. 2002; Zeidán‐Chuliá et al. 2018). However, the role of MMP7 in a demyelinating environment has not been examined. To this end, we investigated whether deletion of MMP7 leads to the formation of fibronectin aggregates and diminished remyelination in the cuprizone model, in which demyelination is not the result of an inflammatory response, but induced through feeding with a toxin that selectively depletes mature oligodendrocytes (Zhan et al. 2020). Achieving a better understanding of the role of individual MMPs is important to devise strategies to remodel the MS lesion environment and stimulate remyelination in MS.

## 2 Methods

### 2.1 Animals and Cuprizone Model

All animal experiments were approved by the Institutional Animal Care and Use Committee at Stony Brook University (federal assurance number A301101) and were performed in compliance with the Guide for the Care and Use of Laboratory Animals of the National Institutes of Health. MMP7 knockout (MMP7^−/−^) mice on a C57BL/6J background were obtained from The Jackson Laboratory (strain number 005111, originally generated by the Matrisian lab; Wilson et al. 1997). Genetic deletion of MMP7 was confirmed by genotyping using primers from the Jackson Laboratory (#12467; #12468; #12413). To induce demyelination, 8‐week‐old male and female MMP7^−/−^ or heterozygous (MMP7^−/+^) mice were fed continuously with a 0.2% cuprizone‐containing diet pellet (Envigo, Madison, Wisconsin) for 3 weeks (acute demyelination), 5 weeks (mixed demyelination/remyelination), or 11 weeks (chronic demyelination). To allow for remyelination, mice were fed with 0.2% cuprizone‐containing chow for 5 weeks, followed by recovery on a normal diet for 1 week (early remyelination) or 2 weeks (remyelination). All cuprizone experiments were executed in the same timeframe, and a single group of 13‐week‐old MMP7^−/+^ or MMP7^−/−^ mice that were only fed regular chow was used as controls for all timepoints. At the designated intervals, the mice were sacrificed and their brains collected for histology, western blot analysis, or electron microscopy.

### 2.2 Tissue Processing

Mice were anesthetized with isoflurane. For histology, mice (*n* = 5–6 per group) were intracardially perfused with ice‐cold phosphate‐buffered saline (PBS, pH 7.4) followed by a freshly prepared ice‐cold solution of 4% paraformaldehyde (PFA, Electron Microscopy Sciences, #30525894) in PBS. The perfusion was performed under RNAse‐free conditions. Brains were removed and fixed in 4% PFA overnight. After cryoprotecting the tissue with 30% sucrose in PBS, the brains were embedded in optical cutting temperature compound (Tissue‐Tek, #4583) and stored at −80°C. Using a cryostat, coronal sections (15 μm) were cut between bregma −0.5 and −2 mm according to the Allen Mouse Brain Atlas (Paxinos and Franklin 2001), the area of the corpus callosum where demyelination is most pronounced (Zhan et al. 2020). Matched sections of MMP7^−/+^ and MMP7^−/−^ were placed on glass slides (VWR Superfrost Plus Adhesion slides, #6310447) and used for immunohistochemistry or in situ hybridization. For protein analysis, the mice (*n* = 5–7 per group) were intracardially perfused with ice‐cold PBS to flush out erythrocytes. The brains were removed and a coronal mouse brain slicer (AgnThos, #6921751) was used to cut three slices of the corpus callosum between approximately 1 and −2.5 bregma. Next, the corpus callosum was manually dissected from the surrounding tissue using a dissection microscope and 22G needles (BD microlance, #304727). Tissue was collected in Eppendorf tubes and one half of the corpus callosum was snap‐frozen for protein analysis and stored at −80°C for downstream analysis. For electron microscopy, mice were intracardially perfused with ice‐cold Rat Ringer's solution (1.27 mM Na_2_HPO_4_, 135 mM NaCl, 5 mM KCl, 1 mM MgCl_2_, 15 mM NaHCO_3_, 2.7 mM CaCl_2_, 1.25% dextrose, pH 7.2) followed by ice‐cold PBS containing 4% PFA and 0.1% fresh electron microscopy grade glutaraldehyde (Sigma‐Aldrich, #G5882). After perfusion, whole brains were removed and stored in 4% PFA, 2% glutaraldehyde in PBS at 4°C until further use.

### 2.3 Histology

#### 2.3.1 Immunofluorescent Labeling and Quantification

Single, double, or triple immunofluorescent labeling was performed to visualize astrocytes (GFAP), proliferating OPCs (Olig2, PDGFRα, Ki67), premyelinating oligodendrocytes (Olig2, BCAS1), and mature oligodendrocytes (Olig2, CC1). For certain epitopes (Table 1), antigen retrieval was performed in 10 mM Tris 1 mM EDTA buffer (pH 9.0) or 10 mM sodium citrate buffer (pH 6.0). Sections were washed three times in PBS. To block nonspecific binding of the secondary antibody, sections were placed in blocking buffer containing 10% goat (Sigma, #G9023) or donkey serum (Sigma‐Aldrich, #D9663) in PBS with 0.1% Triton‐X‐100 (Sigma‐Aldrich, #T9284) for 1 h at room temperature. Next, sections were incubated with primary antibodies (Table 1) overnight at 4°C in a humidified chamber. The following day, sections were washed three times in PBS and incubated with appropriate fluorophore‐conjugated secondary antibodies (Jackson Immunoresearch) for 2 h at room temperature. After three more washes in PBS, nuclei were counterstained with 4′,6‐diamidino‐2‐phenylindole (DAPI; Sigma‐Aldrich, #D5637, 1 μg/mL) and the slides were mounted with SlowFade Gold Antifade mounting medium (Invitrogen, #P36930) or Faramount Aqueous mounting medium (Dako, #S3025). For GFAP, five images spanning the corpus callosum were taken on a Zeiss Axioplan fluorescence microscope at 40× magnification. After equal background subtraction for all images in Adobe Photoshop, the area of GFAP positive pixels was calculated in Fiji (Schindelin et al. 2012) using a predetermined threshold. To quantify the proportions of (proliferating) OPCs and mature oligodendrocytes in the corpus callosum, five images spanning the corpus callosum were taken with a Leica SP8X confocal microscope at 40× magnification. Then, cells were manually counted by a blinded observer using the Fiji cell counter plugin. To quantify the proportions of premyelinating oligodendrocytes, five images were taken on a Leica TCS SP2 AOBS confocal microscope at 40× magnification. Next, images were loaded in Qupath (Bankhead et al. 2017) and the number of BCAS1 positive cells was semi‐automatically determined using a combination of machine learning, as previously described (Rodrigues et al. 2022), followed by manual correction of misclassified cells by a blinded observer.

**TABLE 1:** Primary antibodies.

Target	Company	Antibody #	Species	Type	Dilution for WB	Dilution for IHC	Antigen retrieval
Actin	Sigma‐Aldrich	A5441	Mouse	Monoclonal	1:5000	—	—
Actin	Abcam	AB8227	Rabbit	Polyclonal	1:1000	—	—
BCAS1	Santa Cruz Biotechnology	SC‐136342	Mouse	Monoclonal (IgG1, κ)	—	1:1000	Tris/EDTA buffer, 95°C water bath, 30 min
CC1	Sigma‐Aldrich	OP80	Mouse	Monoclonal (IgG2, b)	—	1:200	Tris/EDTA buffer, 95°C water bath, 30 min
CNP	Sigma‐Aldrich	C5922	Mouse	Monoclonal (IgG1)	1:500	—	—
Fibronectin	Abcam	AB2413	Rabbit	Polyclonal	1:500	—	—
Fibronectin	Chemicon	AB2033	Rabbit	Polyclonal	1:500	—	—
GFAP	Dako	Z0334	Rabbit	Polyclonal	1:5000	1:100	—
IBA1	Wako	019‐19741	Rabbit	Polyclonal	1:1000	1:1000	Citrate buffer, 95°C water bath, 30 min
Ki67	eBioscience	14‐5698‐82	Rat	Monoclonal (IgG2a, κ)	—	1:400	Tris/EDTA buffer, 95°C water bath, 30 min
Laminin 1 + 2	Abcam	AB7463	Rabbit	Polyclonal	1:500	—	—
MBP	Chemicon	MAB386	Rat	Monoclonal, (IgG2a)	1:250	—	—
Olig2	Chemicon	AB9610	Goat	Polyclonal	—	1:200	Tris/EDTA buffer, 95°C water bath, 30 min
PDGFRα	R&D Systems	AF1062	Mouse	Polyclonal	—	1:100	Tris/EDTA buffer, 95°C water bath, 30 min
PLP	Kind gift of Dr. Vijay. Kuchroo, Harvard Medical School, Boston, Massachusetts (Greenfield et al. 2006)	4C2	Mouse	Monoclonal (IgG1)	1:100	—	—

Abbreviations: BCAS1—breast carcinoma amplified sequence 1; CC1—anti‐adenomatous polyposis coli clone 1; CNP—2′,3′‐cyclic nucleotide 3′‐phosphodiesterase; GFAP—glial fibrillary acid protein; IBA1—ionized calcium‐binding adaptor molecule 1; IHC—immunohistochemistry; MBP—myelin basic protein; PDGFRα—platelet‐derived growth factor receptor alpha; PLP—proteolipid protein; WB—western blot.

#### 2.3.2 3,3′‐Diaminobenzidine (DAB) Staining and Quantification

To visualize microglia (IBA1), chromogenic labeling with DAB was performed. After washing and antigen retrieval (Table 1), endogenous peroxidases were blocked by incubating the sections in 0.3% H_2_O_2_ in PBS for 30 min at room temperature in the dark. Next, sections were washed three times in PBS and incubated in blocking buffer (2% normal goat serum in PBS containing 0.1% Triton‐X‐100) for 1 h at room temperature. Sections were incubated overnight with the appropriate primary antibodies (Table 1) in blocking buffer at 4°C in a humidified chamber. The next day, slides were washed three times in PBS. Sections were directly incubated with a horseradish peroxidase‐conjugated secondary antibody (Dako, #P0448) at a 1:100 concentration for 2 h at room temperature. After thorough washing, sections were developed with DAB (Sigma‐Aldrich, #281751) diluted at 1:50 in PBS to which H_2_O_2_ was freshly added to a final concentration of 0.012%. After washing twice in demi water, slides were dehydrated in ascending alcohol concentrations, air‐dried, and then mounted using DePex mounting medium (Serva, #1824301). Slides were imaged using a Hamamatsu Nanozoomer and viewed with NDP.view2 software. To quantify the IBA1 positive area, images of the medial corpus callosum were first exported at 10× magnification. Using Fiji, the corpus callosum was delineated by a blinded observer. Next, images were converted to greyscale and the IBA1 positive area was calculated using a predetermined threshold.

#### 2.3.3 Sudan Black Staining and Quantification

To quantify total myelin levels in the corpus callosum, Sudan Black staining was used. Sections were fixed with 4% PFA in PBS for 10 min and then incubated for 10 min with a pre‐filtered solution of 0.5% Sudan Black B (Sigma‐Aldrich, 199664) in 70% ethanol. After quickly dipping the sections in 70% ethanol to rinse off excess Sudan Black, slides were air‐dried and mounted with Faramount Aqueous mounting medium (Dako, #S3025). Whole slides were imaged using a Hamamatsu Nanozoomer and viewed with NDP.view2 software. Images of the medial corpus callosum were exported at 10× magnification. Using Fiji, the corpus callosum was delineated by a blinded observer, images were converted to greyscale, and the mean pixel intensity was determined (average of two sections).

#### 2.3.4 In Situ Hybridization and Quantification

To label myelin‐producing oligodendrocytes, *c*hromogenic in situ hybridization for proteolipid protein (PLP) was performed as previously described (Chari et al. 2006). Briefly, slides were incubated overnight at 65°C with digoxygenin‐labeled antisense RNA probes (Chari et al. 2006) diluted 1:1000 in hybridization buffer (50% deionized formamide, 10% dextran sulfate, 1× Denhardt's solution, 200 mM NaCl, 5 mM EDTA, 10 mM Tris, 5 mM NaH_2_PO_4_, 5 mM Na_2_HPO_4_, pH 7.5) to which yeast RNA (Sigma‐Aldrich, #R7125) was added at a final concentration of 100 μg/mL. The following day, slides were washed once for 15 min and two times for 30 min at 65°C in washing buffer (50% deionized formamide, 0.1% Tween‐20, 150 mM NaCl, Na_3_C_6_H_5_O_7_, pH 7.0). Next, slides were washed twice for 30 min in maleic salt buffer (0.1% Tween‐20, 100 mM maleic salt, 150 mM NaCl, pH 7.5) at room temperature. After washing, slides were incubated in maleic salt buffer containing 2% blocking reagent (Roche, #1096176) and 10% sheep serum for 1 h at room temperature. Following blocking, slides were incubated with alkaline phosphatase‐conjugated sheep anti‐DIG Fab fragments (1:1500, Roche, #1093274) in blocking solution at 4°C overnight. On the third day, slides were washed three times in maleic salt buffer for 10 min and then incubated twice in predevelopment buffer (100 mM Tris‐HCl, 100 mM NaCl, 50 mM MgCl_2_, pH 9.5) for 10 min. Next, slides were developed in the dark at 37°C in predevelopment buffer to which 1 mM levamisole, 262.5 μg/mL, and 225 μg/mL BCIP were added. After 2 h, the color reaction was stopped by washing in 10 mM Tris and 1 mM EDTA. Slides were dried and mounted with Depex mounting medium (Sigma) containing a drop of xylene. All steps were performed under RNAse‐free conditions. To ensure probe specificity, hybridization with sense probes was performed, which revealed no signal. Whole slides were imaged using a Hamamatsu Nanozoomer and viewed with NDP.view2 software. Per mouse, five images spanning the corpus callosum were exported at 40× magnification. Positive cells were counted by a blinded observer using the ImageJ cell counter plugin.

### 2.4 Western Blotting

Tissues were suspended in 150–300 μL sucrose buffer (0.25 M sucrose, 10 mM Tris, 2 mM EDTA, pH 7.4) containing protease (Roche, #11836170001) and phosphatase inhibitors (Calbiochem, #524625) and homogenized using an electrical potter. Protein concentrations were determined by a detergent‐compatible protein assay (Bio‐Rad, #5000116) using bovine serum albumin as a standard, and the homogenates were stored at −80°C until further use. For western blot analysis, equal amounts of sample (20–25 μg) were diluted in 25% protein sample loading buffer (LI‐COR, #92840004) with a final concentration of 1% sodium dodecyl sulfate (SDS) to which 2.5% β‐mercaptoethanol was added according to the manufacturer's instructions. Samples were heated for 5 min at 95°C in a heat block. After cooling down to room temperature, samples were loaded onto 8% or 12.5% sodium dodecyl sulfate–polyacrylamide (SDS‐PAGE) gels and subjected to western blot analysis under reducing conditions. Proteins were transferred to PVDF membranes (Merk Millipore, Immobilon FL, #IPFL00010) using a semidry or wet blotting system. After washing with PBS and blocking with 50% Intercept PBS blocking buffer (LI‐COR, #92770001) in PBS for 1 h at room temperature, the membranes were incubated overnight at 4°C with primary antibodies (Table 1) diluted in 50% intercept PBS blocking buffer in PBS containing 0.1% Tween (PBS‐T). The next day, the membranes were washed three times with PBS‐T, and then the appropriate IRDye‐conjugated antibodies (LI‐COR), diluted at a 1:25,000 concentration in PBS‐T, were applied for 1 h at room temperature. After thorough washing in PBS‐T, membranes were imaged using an Odyssey CLx Imaging System and quantified with Image Studio Lite software (version 5.2). MMP7^−/+^ and MMP7^−/−^ samples from the various timepoints (control, acute demyelination, mixed demyelination/remyelination, early remyelination, remyelination, chronic demyelination) were loaded on 15‐slot gels. To allow quantitative comparisons between different timepoints, a reference sample was taken along on all gels and set to 1.

### 2.5 Analysis of DOC‐Soluble and DOC‐Insoluble Fibronectin

#### 2.5.1 DOC Extraction

To separate DOC‐soluble and DOC‐insoluble fibronectin, equal amounts of protein (75 μg) were extracted in 100 μL DOC buffer (final concentration: 2% DOC, 5 mM Tris, 2 mM EDTA, pH 8.0) based on a previously published protocol (Wierzbicka‐Patynowski et al. 2004). Samples were stored on ice for 1 h, with intermittent vortexing every 15 min, and were then centrifuged at 16,000*g* for 1 h at 16°C. After the centrifuging step, 75 μL of the DOC‐soluble fraction was carefully taken off and stored in Eppendorf tubes for further processing. For the DOC‐insoluble fraction, the pellet was first washed by adding 75 μL of fresh DOC buffer and centrifuging for an additional 5 min at 16,000*g* at 16°C. After taking off the top 75 μL and adding this to the DOC‐soluble fraction, the remaining buffer was carefully removed, and the DOC‐insoluble pellet was resuspended by vigorously pipetting in 50% protein loading buffer (LI‐COR, #92840004) with a final concentration of 2% SDS. DOC‐soluble and DOC‐insoluble samples were incubated at 37°C for 1 h to allow the association of proteins with SDS. After cooling down to room temperature, samples were loaded onto 8% SDS‐PAGE gels and subjected to nonreducing western blot analysis. After the transfer of proteins using a wet blotting system, membranes were further processed as described above.

### 2.6 Hyaluronan Assay

The commercially available Hyaluronan Quantikine ELISA Kit (R&D Systems, DHYAL0) was used to determine the absolute concentrations of hyaluronan in the corpus callosum according to the manufacturer's instructions using 1 μg of protein per sample (corpus callosum homogenate). The kit is able to detect hyaluronan saccharide chains with a molecular weight of 35 kDa or higher.

### 2.7 Electron Microscopy

Using a brain slicer, coronal sections were cut between −1 and −2 bregma, and small rectangles of the medial corpus callosum were cut out and processed as previously described (Oost et al. 2023). In short, samples were washed with 0.1 M cacodylate buffer (pH 7.4, Sigma‐Aldrich, #20840) before postfixing in 1% osmium tetroxide (Electron Microscopy Sciences, #19114) with 1.5% potassium ferrocyanide (Merck, #P9387) in 0.1 M cacodylate buffer for 2 h at 4°C. Then, tissues were washed in ultrapure water, ascending ethanol concentrations, acetone, and ascending concentrations of epoxy resin (glycid ether 100, SERVA, #21045; 2‐dodecenylsuccinic acid anhydride, SERVA, #20755; methylnadic anhydride, SERVA, #29452; DMP‐30, Polysciences, #00553) prior to embedding in epoxy resin. Using an ultramicrotome and a diamond knife (Diatome, ultra 45°), ultrathin sections (80 nm) were cut of the corpus callosum with the axons in a transversal direction. Sections were placed on formvar‐coated copper L2x1 grids (Agar Scientific, #AGG2500C) and contrasted with 4% neodymium (III) acetate (Sigma‐Aldrich, #325805) in MiliQ (Kuipers and Giepmans 2020). Using a Zeiss Supra 55 scanning electron microscope, images were acquired with a scanning transmission electron microscopy detector connected to an external scan generator ATLAS 5 (Fibics, Ottawa, Canada) at 28/25 kV with a 2.5 nm pixel size. Tiles were stitched together using large‐scale electron microscopy viewer software and uploaded to a nanotomy server (www.nanotomy.org) for viewing. Per animal, five randomly selected areas of 7 by 7 μm were exported at 5.0 nm per pixel. Using the publicly available program MyelTracer (Kaiser et al. 2021), the axons, inner tongues, and myelin sheaths were semi‐automatically delineated (minimum of 125 axons per animal) and the number of myelinated axons was counted by a blinded observer as previously described (Kaiser et al. 2021). The *g*‐ratio was calculated with the formula √(inner myelin area/outer myelin area).

### 2.8 Statistics

Statistical testing was performed with GraphPad Prism 9. Values were assumed to be equally distributed, and the *F*‐test, Brown‐Forsythe's test, and Bartlett's test were used to test for unequal variance. To assess changes within the MMP7^−/+^ or MMP7^−/−^ mice groups between the different timepoints, statistical analyses were performed using one‐way ANOVA followed by Tukey's multiple comparisons test. In case of unequal variance, Welch's ANOVA test was used, followed by Dunnett's T3 multiple comparisons test. To assess changes between MMP7^−/+^ and MMP7^−/−^ mice for each timepoint, unpaired *T*‐tests or, in case of unequal variance, unpaired *T*‐tests with Welch's correction were used. For regression analysis of the electron microscopy experiment, *g*‐ratio was plotted against axon diameter, and the best‐fitting nonlinear line was calculated in GraphPad using least squares regression. The best‐fit parameters of the *Y*‐intercept and slope of the lines for the MMP7^−/+^ and MMP7^−/−^ mice were compared using the extra sum‐of‐squares *F*‐test. In all cases, *p* values of < 0.05, < 0.01, < 0.001, and < 0.0001 were considered significant and are between timepoints indicated with *, **, ***, or **** and between MMP7^−/+^ and MMP7^−/−^ mice with ^#^, ^##^, ^###^, or ^####^, respectively.

## 3 Results

### 3.1 Cuprizone Feeding in MMP7 Deficient Mice Leads to Marked Oligodendrocyte Loss and Demyelination

To better understand the contribution of MMP7 to fibronectin clearance upon demyelination, we employed the cuprizone model in MMP7‐deficient (MMP7^−/−^) mice. As MMP7 has a wide array of substrates, including myelin basic protein (MBP) and myelin‐associated glycoprotein (MAG), both components of the myelin sheath (Chandler et al. 1995; Milward et al. 2008), we first established whether MMP7^−/−^ mice underwent cuprizone‐mediated demyelination.

**FIGURE 1:**
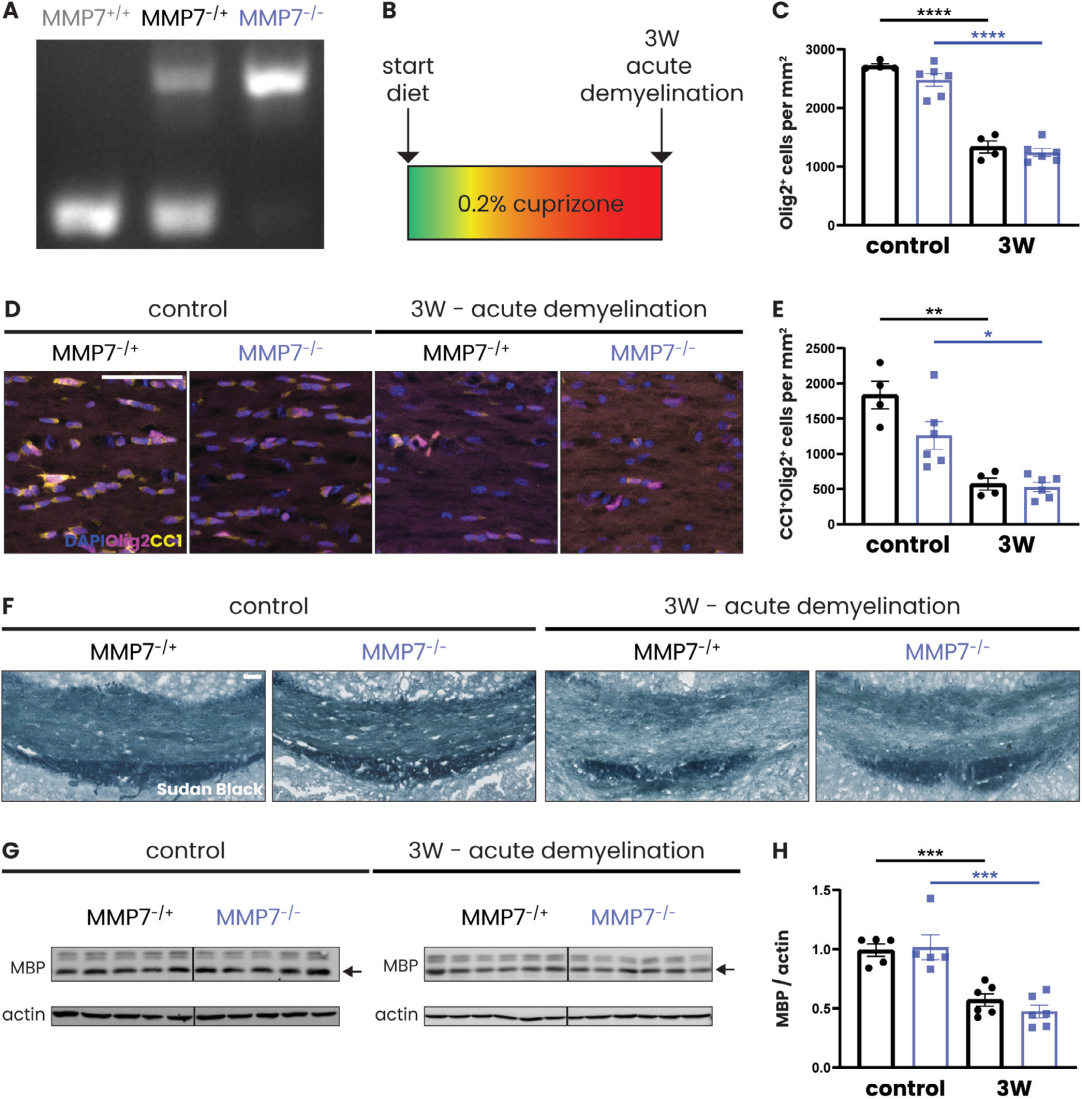
Cuprizone feeding leads to oligodendrocyte loss and demyelination in the corpus callosum of both MMP7^−/+^ and MMP7^−/−^ mice. (A) Genotyping results of wildtype (MMP7^+/+^), heterozygous (MMP7^+/−^), and knockout (MMP7^−/−^) mice. (B) Experimental set‐up: 8‐week‐old male and female MMP7^−/+^ and MMP7^−/−^ mice were fed with 0.2% cuprizone‐containing chow for 3 weeks (acute demyelination). Across all cuprizone experiments, the same group of 13‐week‐old MMP7^−/+^ or MMP7^−/−^ mice fed with regular chow served as controls. (C) Quantification of Olig2^+^ cells in the corpus callosum. (D) Representative images of immunofluorescent staining of the corpus callosum for Olig2 (pink) and CC1 (yellow). Nuclei were visualized with DAPI (blue). (E) Quantification of CC1^+^Olig2^+^ cells in the corpus callosum. (F) Representative images of Sudan Black staining of the corpus callosum. (G) Representative western blots depicting MBP (14 kDa, black arrows) protein levels in the corpus callosum. (H) Quantification of protein levels of the 14 kDa isoform of MBP, which is the most abundant isoform of MBP in the rodent brain (Harauz and Boggs 2013), in the corpus callosum. Values were normalized to actin and visualized relative to a reference sample, which was taken along on each blot and set to 1 (not shown). Data are presented as mean ± SEM. MMP7^−/+^ mice are represented as black dots and MMP7^−/−^ mice as blue squares. To assess changes between different timepoints and between MMP7^−/+^ and MMP7^−/−^ mice (not significant), statistical analyses were performed using unpaired *T*‐tests. In case of unequal variance, unpaired *T*‐tests with Welch's correction was used. Significant data between timepoints are presented (*p < 0.05, **p < 0.01, ***p < 0.001, ****p < 0.0001). Scale bars are 50 μm.

Mice with global deletion of MMP7 (Figure 1A) are viable and do not show gross abnormalities of the brain or other organs (Haro et al. 2000; Page‐McCaw et al. 2007). MMP7 heterozygous (MMP7^−/+^) mice served as controls. MMP7 is endogenously expressed as an inactive enzyme (proMMP7) in the brain (Anthony et al. 1997; Wang et al. 2018; Cossins et al. 1997), indicating that upon injury its activation is regulated by proteases, such as MMP3 (Imai et al. 1995) rather than at the transcriptional level (Wang et al. 2018; Skuljec et al. 2011). Hence, one gene copy likely provides sufficient levels of endogenous proMMP7 compared to wild type.

To induce acute demyelination, 8‐week‐old male and female MMP7^−/+^ and MMP7^−/−^ mice were fed 0.2% cuprizone‐containing chow for 3 weeks (Figure 1B). The extent of demyelination was evaluated in the corpus callosum, where cuprizone‐induced demyelination is most pronounced (Zhan et al. 2020). Consistent with previous reports (Zhan et al. 2020), after 3 weeks of cuprizone feeding, there was a significant loss of oligodendrocyte lineage cells (Figure 1C,D) and mature CC1^+^ oligodendrocytes (Figure 1D,E) in MMP7^−/+^ as well as MMP7^−/−^ mice. This loss of oligodendrocytes resulted in profound demyelination of the corpus callosum, as demonstrated by Sudan Black staining (Figure 1F), which detects fatty substances such as myelin (Ineichen et al. 2017), and by western blot analysis for the myelin‐specific protein MBP (Figure 1G,H). Overall, acute demyelination occurs in the cuprizone model independent of MMP7 expression.

### 3.2 Cuprizone‐Induced Demyelination in MMP7 Deficient Mice Is Accompanied by Microglia and Astrocyte Reactivity

**FIGURE 2:**
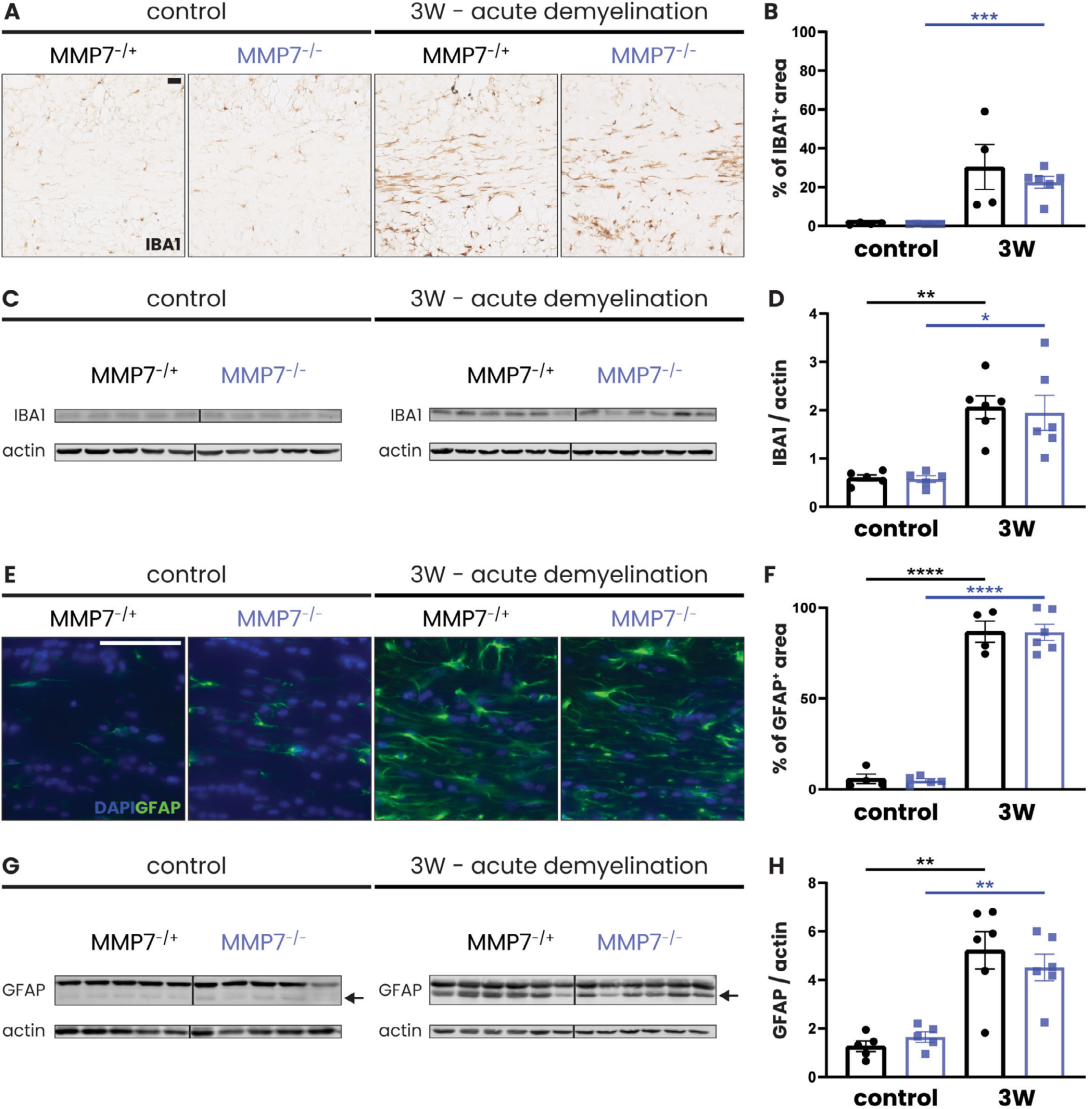
Cuprizone feeding increases microglia and astrocyte reactivity in both MMP7^−/+^ and MMP7^−/−^ mice. Eight‐week‐old male and female MMP7^−/+^ and MMP7^−/−^ mice were fed with 0.2% cuprizone‐containing chow for 3 weeks (acute demyelination). Across all cuprizone experiments, the same group of 13‐week‐old MMP7^−/+^ or MMP7^−/−^ mice fed with regular chow served as controls. (A) Representative images of DAB staining of the corpus callosum for IBA1 (brown). (B) Quantification of IBA1^+^ area in the corpus callosum. (C) Representative western blots depicting IBA1 protein levels in the corpus callosum. (D) Quantification of IBA1 protein levels in the corpus callosum normalized to actin and relative to a reference sample, which was taken along on each blot and set to 1 (not shown). (E) Representative images of immunofluorescent staining of the corpus callosum for GFAP (green). Nuclei were visualized with DAPI (blue). (F) Quantification of GFAP^+^ area in the corpus callosum. (G) Representative western blots depicting GFAP protein levels in the corpus callosum. (H) Quantification of GFAP protein (44 kDa) levels in the corpus callosum normalized to actin and relative to a reference sample, which was taken along on each blot and set to 1 (not shown). Data are presented as mean ± SEM. MMP7^−/+^ mice are represented as black dots and MMP7^−/−^ mice as blue squares. To assess changes between different timepoints and between MMP7^−/+^ and MMP7^−/−^ mice (not significant), statistical analyses were performed using unpaired *T*‐tests. In case of unequal variance, unpaired *T*‐tests with Welch's correction was used. Significant data between timepoints are presented (*p < 0.05, **p < 0.01, ***p < 0.001, ****p < 0.0001). Scale bars are 50 μm.

In the cuprizone model, demyelination coincides with marked activation of microglia and astrocytes (Zhan et al. 2020). To establish whether this reactive response also occurs in the absence of MMP7, we compared the levels of IBA1, a marker for microglia, and GFAP, a marker for most reactive astrocytes, in the corpus callosum of MMP7^−/+^ and MMP7^−/−^ mice (Figure 2A–H). In both experimental groups, cuprizone feeding led to an increase in the immunoreactivity of IBA1 and GFAP (Figure 2A,B,E,F). In addition, total protein levels of IBA1 were equally upregulated in MMP7^−/+^ and MMP7^−/−^ mice (Figure 2C,D), while the 44 kDa isoform of GFAP, which is induced upon cuprizone initiation, also increased in both experimental groups (Figure 2G,H). Therefore, the absence of MMP7 does not markedly impair the microglia and astrocyte response to acute cuprizone‐induced demyelination.

### 3.3 OPC Differentiation Is Not Markedly Impaired in MMP7 Deficient Mice

The combined findings on demyelination as well as microglia and astrocyte reactivity led us to conclude that acute cuprizone‐induced demyelination is unaffected by MMP7 expression, making the cuprizone model a valid method for studying the effects of MMP7 deletion following a demyelinating injury.

**FIGURE 3:**
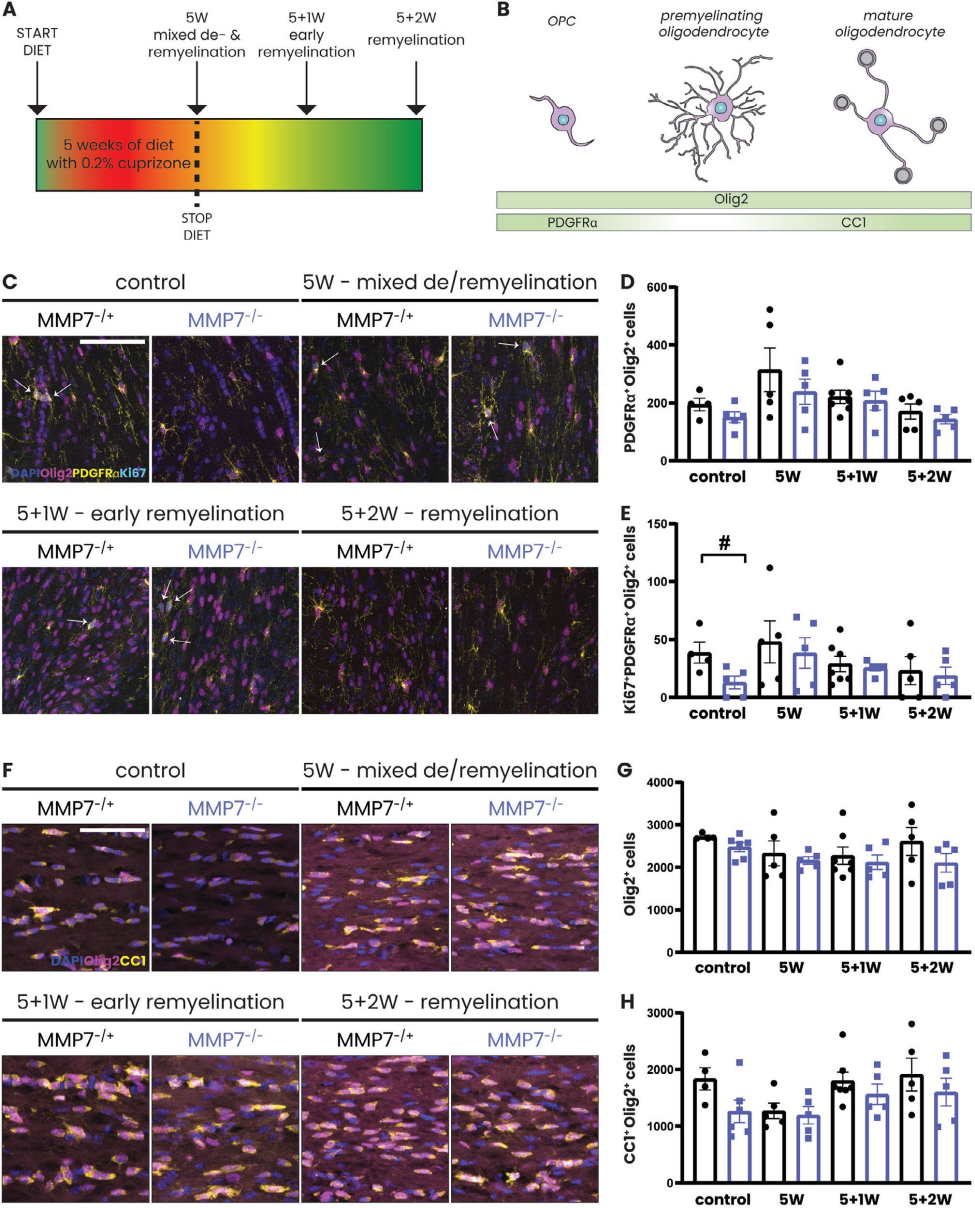
Absence of MMP7 does not markedly impair OPC differentiation. (A) Experimental set‐up: 8‐week‐old male and female MMP7^−/+^ and MMP7^−/−^ mice were fed with 0.2% cuprizone‐containing chow for 5 weeks (mixed demyelination/remyelination) or with 0.2% cuprizone for 5 weeks followed by recovery on a normal diet for 1 week (early remyelination) or 2 weeks (remyelination). Across all cuprizone experiments, the same group of 13‐week‐old MMP7^−/+^ or MMP7^−/−^ mice fed with regular chow served as controls. (B) Schematic representation of the presence of Olig2 (oligodendrocyte lineage marker), PDGFRα (OPC marker), and CC1 (mature oligodendrocyte marker) during oligodendrocyte maturation. (C) Representative images of immunofluorescent staining of the corpus callosum for Olig2 (pink), PDGFRα (yellow), and Ki67 (cyan). Nuclei were visualized with DAPI (blue). Proliferating OPCs are highlighted with white arrows. (D) Quantification of PDGFRα^+^Olig2^+^ cells in the corpus callosum. (E) Quantification of Ki67^+^PDGFRα^+^ Olig2^+^ cells in the corpus callosum. (F) Representative images of immunofluorescent staining of the corpus callosum for Olig2 (pink) and CC1 (yellow). Nuclei were visualized with DAPI (blue). (G) Quantification of Olig2^+^ cells in the corpus callosum. (H) Quantification of CC1^+^Olig2^+^ cells in the corpus callosum. Data are presented as mean ± SEM. MMP7^−/+^ mice are represented as black dots and MMP7^−/−^ mice as blue squares. To assess changes between different timepoints, statistical analyses were performed using one‐way ANOVA followed by Tukey's multiple comparisons test (not significant). In case of unequal variance, Welch's ANOVA test followed by Dunnet's T3 multiple comparisons test was used. To assess changes between MMP7^−/+^ and MMP7^−/−^ mice, unpaired *T*‐tests or, in case of unequal variance, unpaired *T*‐tests with Welch's correction were used. Significant data between MMP7^−/+^ and MMP7^−/−^ mice are presented (^#^ *p* < 0.05). Scale bars are 50 μm.

Accordingly, MMP7^−/+^ and MMP7^−/−^ mice were fed cuprizone (Figure 3A) for 5 weeks (mixed demyelination/remyelination). At this timepoint, demyelination is at its height, but simultaneously proliferating OPCs have started to repopulate the corpus callosum in response to the cuprizone‐induced oligodendrocyte loss (Zhan et al. 2020). When, after 5 weeks, mice are returned to a normal diet (Figure 3A), the OPCs differentiate into myelinating oligodendrocytes, and myelin levels start to recover within 1 week (early remyelination) and are almost back to normal by 2 weeks (remyelination).

To assess the extent of OPC proliferation and differentiation in the absence of MMP7, sections of the corpus callosum were stained for markers expressed at different stages of the oligodendrocyte life cycle (Fard et al. 2017) (Figure 3B). Pre‐cuprizone, the number of proliferating OPCs, defined by immunopositivity for Olig2, PDGFRα, and Ki67, was significantly lower in MMP7^−/−^ compared to MMP7^−/+^ mice (Figure 3C–E). However, this subtle difference had caught up by 5 weeks of cuprizone diet (Figure 3E) and was not accompanied by significant alterations in the numbers of PDGFRα^+^ OPCs (Figure 3D) or total numbers of Olig2^+^ oligodendrocyte lineage cells (Figure 3F,G) at any of the analyzed timepoints. To quantify OPC differentiation, we stained for CC1, a mature oligodendrocyte marker, hypothesizing that in MMP7^−/−^ mice, OPC differentiation would be impaired (Figure 3F–H). However, contrary to expectation, we observed no differences between the genotypes in CC1^+^ oligodendrocyte numbers before or after cuprizone withdrawal (Figure 5G). Additional immunohistochemistry for BCAS1, a marker for premyelinating oligodendrocytes (Fard et al. 2017), confirmed that OPC differentiation was not delayed in the absence of MMP7 (Figure S1A–D). Of note, in situ hybridization for *PLP* mRNA, as a measure for actively myelin‐producing cells, showed that *PLP* mRNA^+^ cell numbers were significantly decreased in the corpus callosum of MMP7^−/−^ mice after 5 weeks of cuprizone (Figure S1E,F). However, this effect had already recovered 1 week following cuprizone cessation, indicating that, if anything, the reduction in *PLP* mRNA expressing cells represents only a transient delay in OPC differentiation in the absence of MMP7. Overall, we conclude that the lack of MMP7 does not markedly impair OPC differentiation following acute cuprizone‐induced demyelination, although the temporal dynamics of OPC differentiation appear to be subtly altered.

### 3.4 Remyelination Proceeds Efficiently in MMP7 Deficient Mice

**FIGURE 4:**
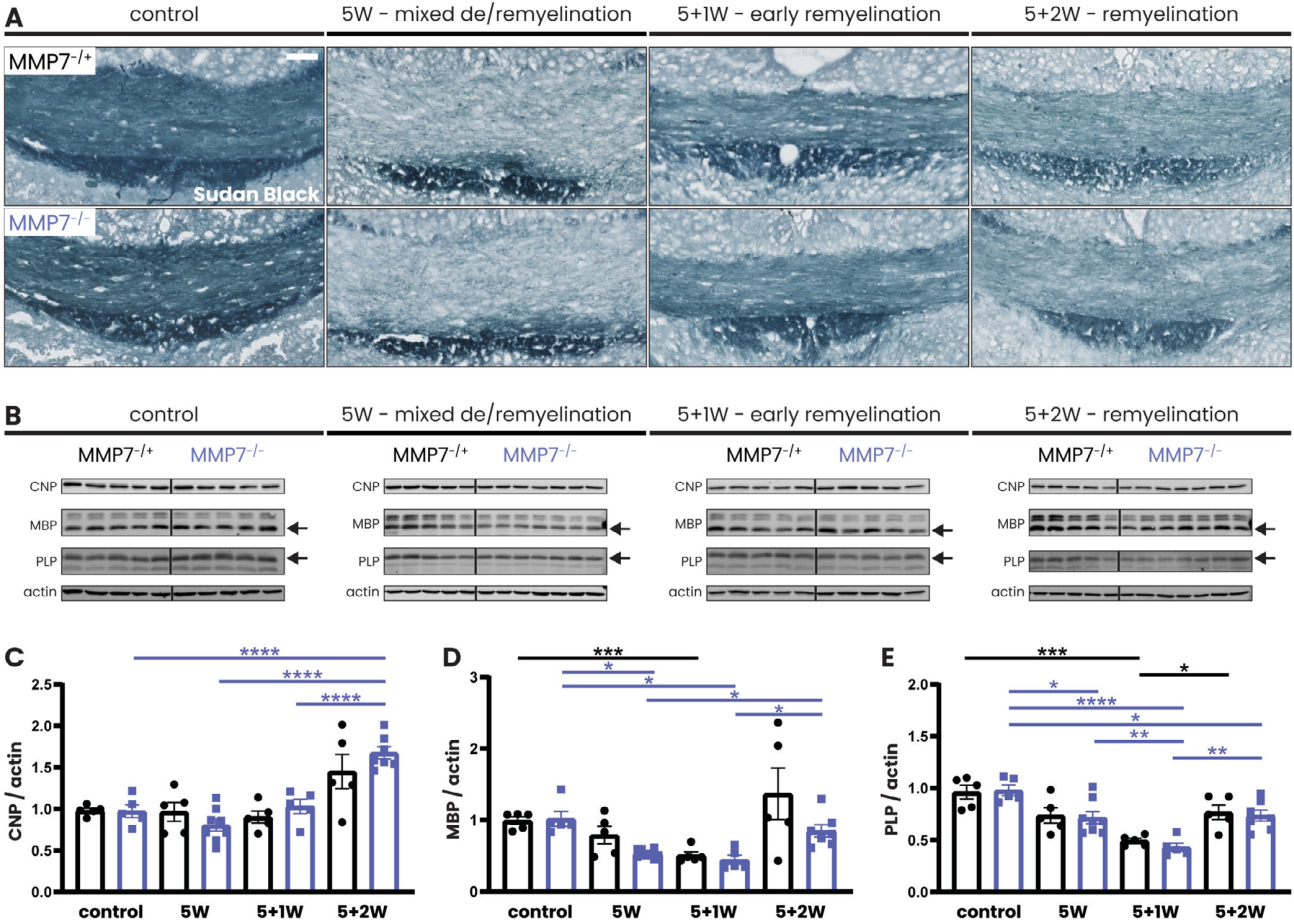
Myelin protein levels recover in equal measure in MMP7^−/+^ and MMP7^−/−^ mice. Eight‐week‐old male and female MMP7^−/+^ and MMP7^−/−^ mice were fed with 0.2% cuprizone‐containing chow for 5 weeks (mixed demyelination/remyelination) or with 0.2% cuprizone for 5 weeks followed by recovery on a normal diet for 1 week (early remyelination) or 2 weeks (remyelination). Across all cuprizone experiments, the same group of 13‐week‐old MMP7^−/+^ or MMP7^−/−^ mice fed with regular chow served as controls. (A) Representative images of Sudan Black staining of the corpus callosum. (B) Representative western blots depicting CNP (46 kDa), PLP (30 kDa, black arrows), and MBP (14 kDa, black arrows) protein levels in the corpus callosum. (C–E) Quantification of CNP (46 kDa), PLP (30 kDa), and MBP (14 kDa) protein levels in the corpus callosum normalized to actin and relative to a reference sample, which was taken along on each blot and set to 1 (not shown). Data are presented as mean ± SEM. MMP7^−/+^ mice are represented as black dots and MMP7^−/−^ mice as blue squares. To assess changes between different timepoints, statistical analyses were performed using one‐way ANOVA followed by Tukey's multiple comparisons test. In case of unequal variance, Welch's ANOVA test followed by Dunnet's T3 multiple comparisons test was used. To assess changes between MMP7^−/+^ and MMP7^−/−^ mice, unpaired *T*‐tests or, in case of unequal variance, unpaired *T*‐tests with Welch's correction were used (not significant). Significant data between timepoints are presented (*p < 0.05, **p < 0.01, ***p < 0.001, ****p < 0.0001). Scale bars are 50 μm.

**FIGURE 5:**
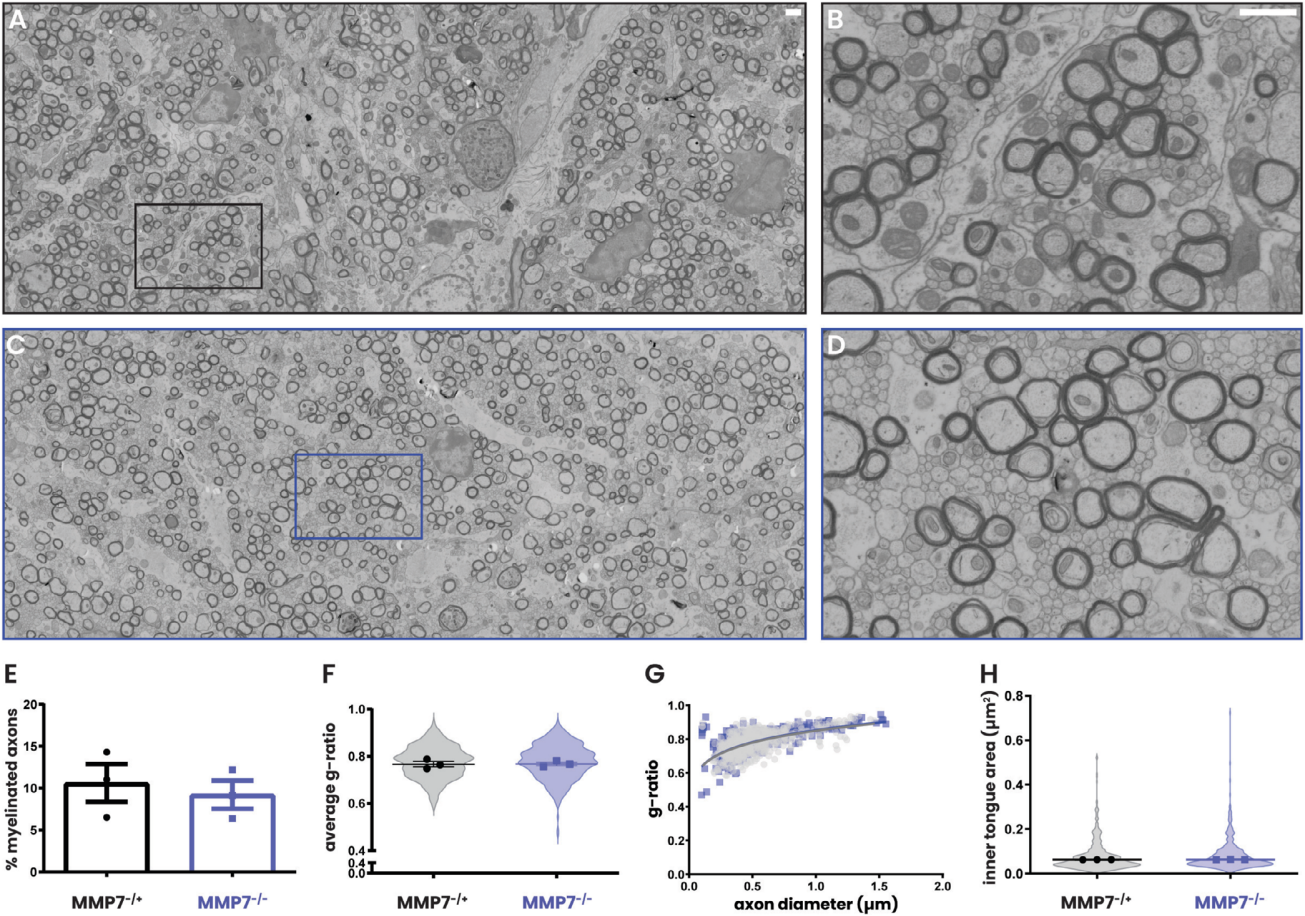
Two weeks post‐cuprizone cessation, remyelination is equal in MMP7^−/+^ and MMP7^−/−^ mice at the ultrastructural level. Eight‐week‐old male and female MMP7^−/+^ and MMP7^−/−^ mice were fed with 0.2% cuprizone‐containing chow for 5 weeks followed by recovery on a normal diet for 2 weeks. (A–D) Representative electron microscopy images of the corpus callosum of (A and B) MMP7^−/+^ and (C and D) MMP7^−/−^ mice. (E) Quantification of the percentage of total myelinated axons in the corpus callosum. (F) Violin plots depicting the *g*‐ratio distribution of all measured myelinated axons in the corpus callosum; data points represent average *g*‐ratio per mice. (G) Regression data of *g*‐ratio on axon diameter in the corpus callosum. (H) Violin plots depicting the inner tongue area distribution of all measured myelinated axons in the corpus callosum; data points represent average inner tongue area per mice. MMP7^−/+^ mice are represented as black dots and MMP7^−/−^ mice as blue squares. For the nonlinear regression analysis, extra sum‐of‐squares *F*‐test was used to compare the *Y*‐intersect and slope of the best nonlinear fitted curve of the two datasets (not significant). For other analyses, data are presented as mean ± SEM. Changes between MMP7^−/+^ and MMP7^−/−^ mice were assessed using unpaired *T*‐tests or, in case of unequal variance, unpaired *T*‐tests with Welch's correction (not significant). Scale bars are 1 μm.

As OPC differentiation was not markedly impaired, we next asked whether the newly differentiated oligodendrocytes were capable of efficient remyelination in the absence of MMP7. To this end, we assessed remyelination in the corpus callosum at 1 and 2 weeks post‐cuprizone cessation. First, myelin was visualized using Sudan Black staining, which revealed a similar pattern of efficient remyelination in both genotypes (Figure 4A). Moreover, examination of total myelin protein expression with western blotting revealed that levels of CNP, PLP, and MBP (Figure 4B–E) recovered equally in MMP7^−/+^ and MMP7^−/−^ mice. Lastly, electron microscopy was used to compare the amount of remyelinated axons in the corpus callosum of MMP7^−/+^ (Figure 5A,B) and MMP7^−/−^ mice (Figure 5C,D) after 2 weeks of cuprizone cessation (remyelination). No differences were found between MMP7^−/+^ and MMP7^−/−^ mice in the percentage of myelinated axons (Figure 5E), *g*‐ratio (Figure 5F,G), nor the thickness of the inner tongue (Figure 5H), a reflection of the degree of myelin compaction. In conclusion, following acute cuprizone‐induced demyelination, there is efficient remyelination in the absence of MMP7.

### 3.5 In MMP7 Deficient Mice, Fibronectin Aggregates Do Not Accumulate in the Remyelination Phase

Initially, we hypothesized that a lack of MMP7 would impair fibronectin clearance following cuprizone‐induced demyelination, leading to the formation of remyelination‐inhibiting fibronectin aggregates. As OPC differentiation and remyelination efficiency were not altered in the absence of MMP7, we next asked whether this was due to a lack of fibronectin aggregate formation in the remyelination phase.

**FIGURE 6:**
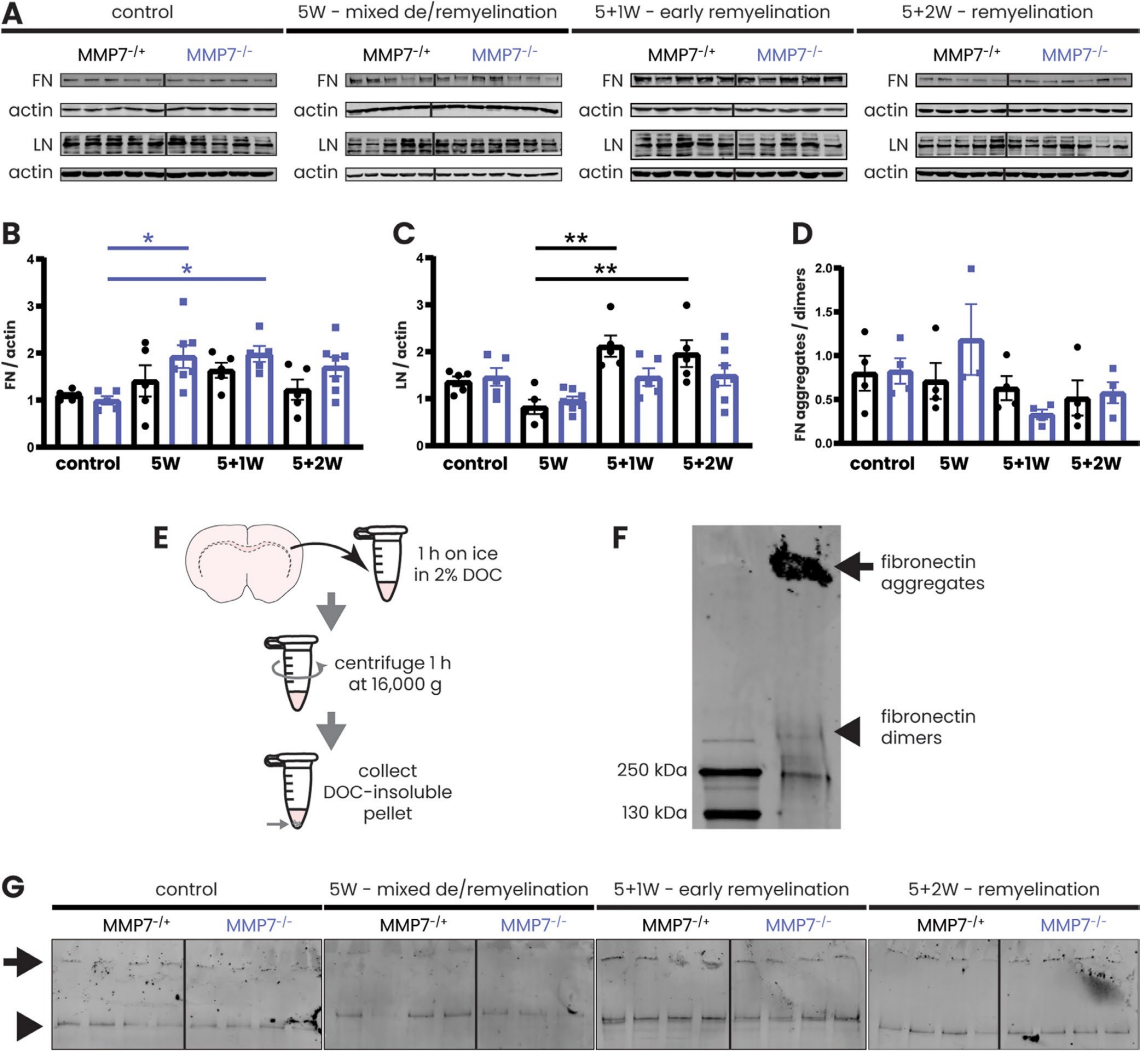
During remyelination, absence of MMP7 does not lead to accumulation of ECM proteins, including fibronectin aggregates. Eight‐week‐old male and female MMP7^−/+^ and MMP7^−/−^ mice were fed with 0.2% cuprizone‐containing chow for 5 weeks (mixed demyelination/remyelination) or with 0.2% cuprizone for 5 weeks followed by recovery on a normal diet for 1 week (early remyelination) or 2 weeks (remyelination). Across all cuprizone experiments, the same group of 13‐week‐old MMP7^−/+^ or MMP7^−/−^ mice fed with regular chow served as controls. (A) Representative western blots depicting fibronectin and laminin protein levels in the corpus callosum. (B) Quantification of total fibronectin protein levels in the corpus callosum normalized to actin and relative to a reference sample, which was taken along on each blot and set to 1 (not shown). (C) Quantification of laminin protein levels in the corpus callosum normalized to actin and relative to a reference sample, which was taken along on each blot and set to 1 (not shown). (D) Quantification of the fibronectin aggregate to dimer ratio in the DOC‐insoluble fraction. (E) Schematic representation of the workflow for extracting DOC‐insoluble proteins from corpus callosum homogenates. (F) Example of primary rat astrocyte‐derived fibronectin aggregates accumulating in the stacking gel on nonreducing western blot. Black arrows indicate the DOC‐insoluble fibronectin aggregates (stacking gel) and black arrowheads indicate the fibronectin dimers (440 kDa). (G) Representative nonreducing western blots depicting the levels of DOC‐insoluble fibronectin aggregates (arrow) and fibronectin dimers (arrowhead) in the corpus callosum. Data are presented as mean ± SEM. MMP7^−/+^ mice are represented as black dots and MMP7^−/−^ mice as blue squares. To assess changes between different timepoints, statistical analyses were performed using one‐way ANOVA followed by Tukey's multiple comparisons test. In case of unequal variance, Welch's ANOVA test followed by Dunnet's T3 multiple comparisons test was used. To assess changes between MMP7^−/+^ and MMP7^−/−^ mice, unpaired *T*‐tests or, in case of unequal variance, unpaired *T*‐tests with Welch's correction were used (not significant). Significant data between timepoints are presented (*p < 0.05, **p < 0.01). FN—fibronectin; LN—laminin.

Compared to the non‐cuprizone control, total fibronectin levels were significantly increased up to twofold in MMP7^−/−^ mice at 5 weeks of cuprizone feeding as well as 1 week post‐cuprizone cessation (Figure 6A,B). This effect was not seen in the MMP7^−/+^ group (Figure 6A,B), suggesting a somewhat steeper increase in fibronectin levels in the absence of MMP7. However, at none of these timepoints did the absolute levels differ significantly between MMP7^−/+^ and MMP7^−/−^ mice (Figure 6B). Besides fibronectin, levels of laminin, another MMP7 substrate, were analyzed, and similarly, the absence of MMP7 did not lead to changes in laminin levels (Figure 6C,D). Lastly, when DOC‐soluble proteins were separated from DOC‐insoluble proteins and the insoluble fraction was subjected to nonreducing western blotting (Figure 6E,F), the amount of fibronectin aggregates that accumulated in the stacking gel was overall negligible and not increased in MMP7^−/−^ compared to MMP7^−/+^ animals (Figure 6G,H). Together, these findings indicate that the absence of MMP7 in the cuprizone model does not lead to the accumulation of fibronectin (aggregates) or laminin following acute cuprizone‐induced demyelination.

### 3.6 MMP7 Deficient Mice Have Lower Levels of Fibronectin and Hyaluronan After Chronic Demyelination

**FIGURE 7:**
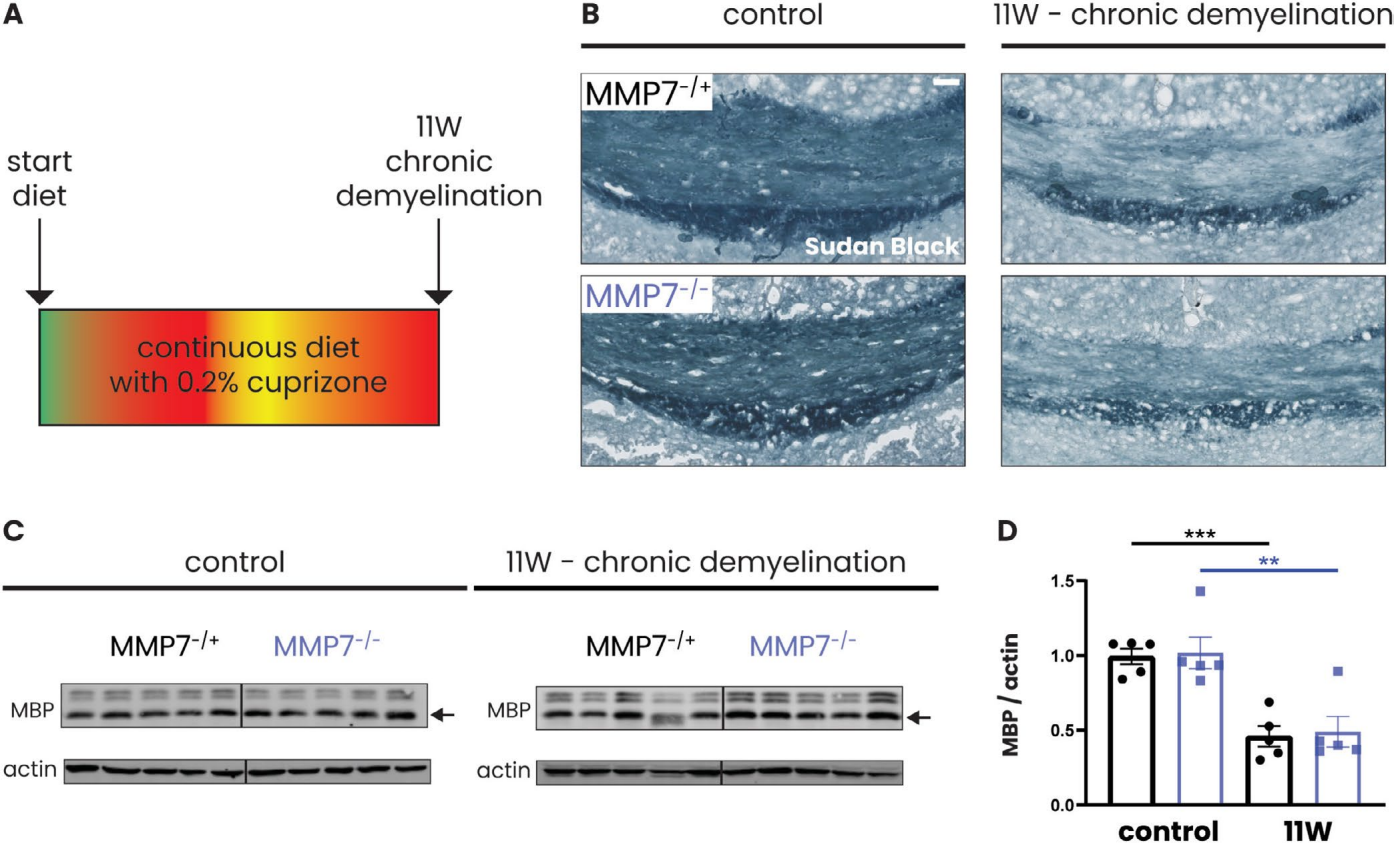
Chronic cuprizone feeding leads to progressive demyelination. (A) Experimental set‐up: 8‐week‐old male and female MMP7^−/+^ and MMP7^−/−^ mice were fed with 0.2% cuprizone‐containing chow for 11 weeks (chronic demyelination). Across all cuprizone experiments, the same group of 13‐week‐old MMP7^−/+^ or MMP7^−/−^ mice fed with regular chow served as controls. (B) Representative images of Sudan Black staining of the corpus callosum. (C) Representative western blots depicting MBP (14 kDa, black arrows), CNP, and PLP protein levels in the corpus callosum. (D) Quantification of MBP (14 kDa) levels in the corpus callosum normalized to actin and relative to a reference sample, which was taken along on each blot and set to 1 (not shown). Data are presented as mean ± SEM. MMP7^−/+^ mice are represented as black dots and MMP7^−/−^ mice as blue squares. To assess changes between different timepoints and between MMP7^−/+^ and MMP7^−/−^ mice (not significant), statistical analyses were performed using unpaired *T*‐tests. In case of unequal variance, unpaired *T*‐tests with Welch's correction was used. Significant data between timepoints are presented (**p < 0.01, ***p < 0.001). Scale bars are 50 μm. MBP—myelin basic protein.

**FIGURE 8:**
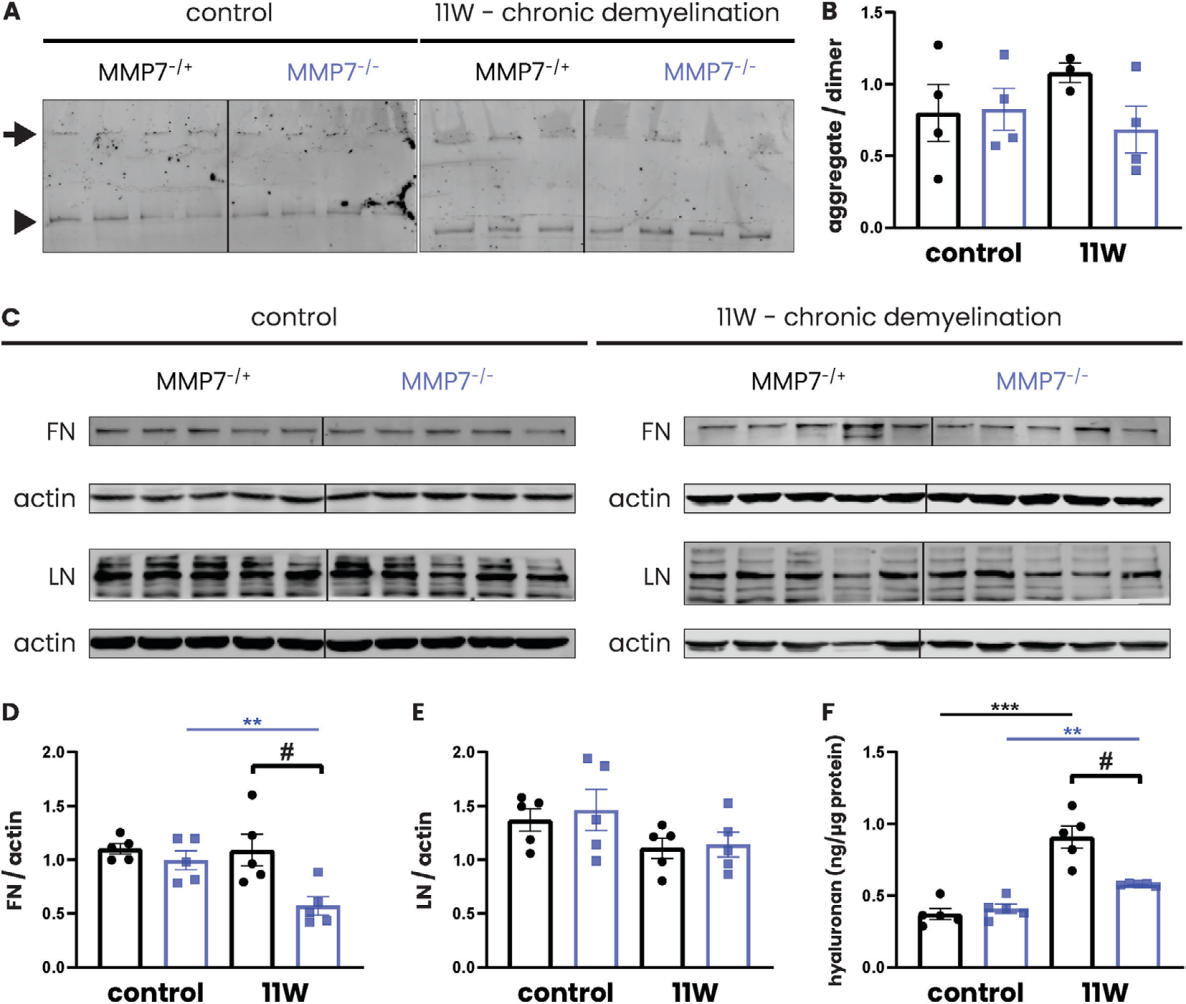
Unexpectedly, absence of MMP7 leads to lower levels of fibronectin and hyaluronan in the setting of chronic demyelination. Eight‐week‐old male and female MMP7^−/+^ and MMP7^−/−^ mice were fed with 0.2% cuprizone‐containing chow for 11 weeks (chronic demyelination). Across all cuprizone experiments, the same group of 13‐week‐old MMP7^−/+^ or MMP7^−/−^ mice fed with regular chow served as controls. (A) Representative nonreducing western blots depicting the levels of DOC‐insoluble fibronectin aggregates (black arrow) and fibronectin dimers (black arrowheads) in the corpus callosum. (B) Quantification of the fibronectin aggregate to dimer ratio. (C) Representative western blots depicting total fibronectin and laminin protein levels in the corpus callosum. (D and E) Quantification of (D) fibronectin and (E) laminin protein levels normalized to actin and relative to a reference sample, which was taken along on each blot and set to 1 (not shown). (F) Quantification of hyaluronan levels in the corpus callosum, determined using a commercially available enzyme‐linked immunosorbent assay. Data are presented as mean ± SEM. MMP7^−/+^ mice are represented as black dots and MMP7^−/−^ mice as blue squares. To assess changes between timepoints and between MMP7^−/+^ and MMP7^−/−^ mice, unpaired *T*‐tests were employed. In case of unequal variance, unpaired *T*‐tests with Welch's correction was used. Significant data between timepoints (**p < 0.01, ***p < 0.001) and between MMP7^−/+^ and MMP7^−/−^ mice (^#^ *p* < 0.05) are presented. FN—fibronectin; LN—laminin.

Thus, while fibronectin aggregates are found in chronic MS lesions and at the height of chronic relapsing EAE (Stoffels, de Jonge, et al. 2013), they are not detected following acute cuprizone‐induced demyelination, even in MMP7^−/−^ mice. Wondering whether this discrepancy could be explained by the different chronicity of these demyelinating insults, we next investigated whether fibronectin aggregates would be formed in MMP7^−/−^ mice after prolonged cuprizone intoxication. Eleven weeks of chronic cuprizone administration (Figure 7A) led to overt demyelination in the corpus callosum of both MMP7^−/+^ and MMP7^−/−^ mice as demonstrated by Sudan Black staining (Figure 7B) and by western blotting for MBP (Figure 7C,D). However, this chronic demyelinating insult did not coincide with the formation of fibronectin aggregates, even in MMP7^−/−^ mice (Figure 8A,B). Contrary to expectation, total fibronectin protein levels were significantly lower in MMP7^−/−^ compared to MMP7^−/+^ mice (Figure 8C,D), while levels of laminin were not altered (Figure 8E). Hyaluronan is an ECM molecule that has previously been implicated in remyelination failure in MS (Back et al. 2005; Sloane et al. 2010), but which is not known to be degraded by MMPs (Sherman et al. 2015). Intriguingly, after 11 weeks of cuprizone, hyaluronan levels were robustly elevated in the corpus callosum of MMP7^−/+^ mice, but this increase was almost negligible in their MMP7^−/−^ counterparts (Figure 8F). At this timepoint, levels of hyaluronan were significantly lower in MMP7^−/−^ compared to the MMP7^−/+^ mice (Figure 8F). Overall, these findings point to an unexpected complexity in how MMP7 influences fibronectin and hyaluronan levels in the setting of chronic demyelination.

## 4 Discussion

The present study employed the cuprizone model to investigate the role of MMP7 in remyelination failure, hypothesizing that MMP7 deficiency would lead to an accumulation of ECM proteins at the onset of remyelination, including fibronectin, leading to the formation of remyelination‐inhibiting fibronectin aggregates. Unexpectedly, remyelination proceeded efficiently in the absence of MMP7. Following cuprizone withdrawal, the lack of MMP7 did not cause the persistence of fibronectin aggregates or of laminin, another MMP7 substrate. In fact, after chronic demyelination, fibronectin levels were surprisingly lower in MMP7^−/−^ mice. Moreover, in the chronic cuprizone model, the ECM molecule hyaluronan, which is not known to be degraded by MMPs (Sherman et al. 2015), showed a pronounced increase in MMP7^−/+^ but not MMP7^−/−^ mice. Overall, these results indicate that MMP7 is not essential for de‐ and remyelination in the cuprizone model and point to an unexpected effect of MMP7 deficiency on fibronectin and hyaluronan levels during chronic demyelination.

In the EAE model, constitutive lack of MMP7 protects from demyelination by preventing the influx of peripheral immune cells (Buhler et al. 2009). However, in the current study, cuprizone‐induced demyelination occurred despite the absence of MMP7. This illustrates that in the cuprizone model, contrary to EAE, demyelination is likely not driven by (MMP7‐mediated) disruption of the blood–brain barrier and infiltration of peripheral immune cells, but a direct consequence of cuprizone‐induced oligodendrocyte death and the subsequent local inflammatory response of CNS‐resident cells (Vega‐Riquer et al. 2019). Hence, in the cuprizone model, lack of MMP7 mainly affects processes initiated upon demyelination.

MMP7 efficiently cleaves a wide array of ECM proteins in vitro (Stoffels, de Jonge, et al. 2013; Imai et al. 1995; Siri et al. 1995; Agnihotri et al. 2001) and in chronic MS lesions, its reduced expression is associated with the presence of fibronectin aggregates (Stoffels, de Jonge, et al. 2013). Nevertheless, in the current study, lack of MMP7 did not result in the accumulation of fibronectin aggregates in the remyelination phase, despite a transient twofold increase in dimeric fibronectin in the MMP7^−/−^ group. On the one hand, this suggests that the lack of MMP7 in chronic MS lesions is not causative for fibronectin aggregate accumulation and remyelination failure. On the other hand, it is possible that in MMP7^−/−^ mice, another protease compensated for the absence of MMP7 following cuprizone‐induced demyelination. Indeed, substrate specificities of MMPs often overlap in vitro (Itoh 2015; Djuric and Zivkovic 2017). Moreover, a number of previous studies exploring the knockout of MMPs in different models of tissue injury have reported redundancy of MMPs in vivo (Nguyen et al. 2020). For example, MMP13 expression is upregulated in cutaneous wounds (Wu et al. 2003; Hartenstein et al. 2006), likely to facilitate the timely degradation of repair‐inhibiting collagens (Liu et al. 1995). However, the deletion of MMP13 does not lead to collagen accumulation nor to impaired wound healing due to compensatory upregulation of MMP8, another collagen‐degrading enzyme (Hartenstein et al. 2006). These findings highlight the complexity of studying the contribution of individual MMPs by using constitutive knockout mice (Nguyen et al. 2020; Rodríguez et al. 2010). Although MMP7 is the only MMP known to degrade fibronectin aggregates (Wang et al. 2018; Bredow et al. 1995), at least 10 other MMPs degrade fibronectin in vitro (Djuric and Zivkovic 2017; de Jong et al. 2020). In addition, other types of proteases, such as a disintegrin and metalloproteinase 8 (ADAM8) (Zack et al. 2009), may contribute to fibronectin clearance. In MMP7^−/−^ mice, compensatory upregulation of any of these enzymes, or a combination thereof, may prevent the accumulation of fibronectin upon cuprizone‐induced demyelination.

Contrary to expectation, fibronectin levels were even lower in MMP7^−/−^ than in MMP7^−/+^ mice after prolonged cuprizone administration. This could point to overcompensation for the lack of MMP7 by another protease or, alternatively, to reduced synthesis of fibronectin. Intriguingly, MMP7 deficiency also prevented hyaluronan accumulation. After 11 weeks of cuprizone diet, levels of hyaluronan were markedly elevated in MMP7^+/−^ but not in MMP7^−/−^ mice. Of note, hyaluronan is not known to be degraded by MMPs, but by a specific group of enzymes called hyaluronidases (Sherman et al. 2015; Žádníková et al. 2022). To our knowledge, there is no single protease that, if elevated to compensate for the absence of MMP7, would degrade both fibronectin and hyaluronan. Therefore, a more likely explanation for the lower fibronectin and hyaluronan levels at chronic demyelination in MMP7 knockout mice would be a non‐ECM cleaving function of MMP7 that either directly or indirectly alters fibronectin and hyaluronan production. In mice, levels of fibronectin and of the ECM protein tenascin C, another MMP7 substrate (Siri et al. 1995), increase in response to myocardial infarction (Konstandin et al. 2013), but counterintuitively, this effect is less pronounced in MMP7 knockout mice (Chiao et al. 2010). Likewise, in response to kidney injury, the extent of fibronectin and collagen deposition is reduced in mice without MMP7 (Zhou et al. 2017). All of this suggests that the permanent absence of MMP7 reduces ECM production following certain types of injury, for example, by preventing MMP7‐mediated activation of pro‐fibrotic E‐cadherin signalling (Zhou et al. 2017) or by reducing the activation of the pro‐inflammatory cytokine TNFα (Haro et al. 2000; Gearing et al. 1994), which in turn regulates the production of hyaluronan by hyaluronan synthases (Tammi et al. 2011).

Although this study indicates that MMP7 is not essential for successful remyelination in the cuprizone model, our findings do not necessarily rule out a role for MMP7 in fibronectin aggregate accumulation in MS lesions. Firstly, the compensatory effects that may occur in the cuprizone model are likely absent from chronic MS lesions, as here the expression of other MMPs, including MMP3 (Wang et al. 2018), is also decreased, while fibronectin aggregates do accumulate. Secondly, besides reduced MMP7 expression, there may be additional triggers present in MS lesions, such as chronic inflammation, that are necessary for fibronectin aggregate formation (Werkman et al. 2020). When astrocytes are stimulated with the Toll‐like receptor 3 agonist Poly I:C, fibronectin aggregates are formed (Werkman et al. 2020). Of note, when astrocytes are pretreated with pro‐inflammatory cytokines, mimicking the chronic inflammatory environment in MS lesions, this response is exacerbated (Werkman et al. 2020). In the cuprizone model, inflammation is limited, and such a “double hit” is likely absent. In contrast, fibronectin aggregates are present in the relapse phase of chronic EAE (Stoffels, de Jonge, et al. 2013). Using this model, future studies could explore whether the inducible knockout of MMP7 (post‐disease onset, as MMP7 is necessary for EAE development; Buhler et al. 2009) would exacerbate the accumulation of fibronectin aggregates in a more inflammatory setting. Lastly, even if reduced MMP7 expression is not involved in the formation of fibronectin aggregates, the therapeutic delivery of MMP7 may still be helpful for their clearance, as to date, MMP7 is the only MMP known to degrade DOC‐insoluble fibronectin aggregates (Wang et al. 2018; Bredow et al. 1995). To establish or eliminate MMP7 as a therapeutic target for fibronectin removal, future studies should focus on models where fibronectin aggregates are known to be formed, such as in chronic relapsing EAE (Stoffels, de Jonge, et al. 2013) and explore whether local delivery of active MMP7, for example, with nanoparticles, promotes remyelination.

In summary, MMP7 is not essential for demyelination and efficient remyelination in the cuprizone model. These findings highlight the complexity of the MMP system (Rodríguez et al. 2010) and suggest that the absence of MMP7 is not the sole factor causing the accumulation of fibronectin aggregates in MS lesions. Indeed, it is likely that in MS, remyelination failure is a complex interplay of aberrant ECM production and a dysregulated ECM clearance network. Determining the individual effects of MMP7 and its redundancy to ECM remodeling during demyelination and remyelination contributes to a better understanding of remyelination failure. Ultimately, this knowledge may help to devise strategies to remodel the MS lesion environment and stimulate remyelination.

## 5 Author Contributions

**Rianne P. Gorter:** formal analysis, funding acquisition, investigation, project administration, visualisation and writing – original draft. **Andrea J. Arreguin:** investigation. **Wendy Oost:** investigation. **Jenny C. de Jonge:** investigation. **Harm H. Kampinga:** supervision, writing – review and editing. **Sandra Amor:** supervision, writing – review and editing. **Holly Colognato:** resources, supervision, project administration, writing – review and editing. **Wia Baron:** conceptualization, funding acquisition, project administration, resources, supervision, writing – review and Editing.

## Conflicts of Interest

The authors declare no conflicts of interest.

## Supporting information


Figure S1.

## Data Availability

The STEM datasets generated and analyzed in the current study are publicly available at full resolution at nanotomy.org. All other data generated during and/or analyzed during the current study are available from the corresponding author on reasonable request.

## References

[glia70005-bib-0001] Agnihotri, R. , H. C. Crawford , H. Haro , L. M. Matrisian , M. C. Havrda , and L. Liaw . 2001. “Osteopontin, a Novel Substrate for Matrix Metalloproteinase‐3 (Stromelysin‐1) and Matrix Metalloproteinase‐7 (Matrilysin).” Journal of Biological Chemistry 276, no. 30: 28261–28267. 10.1074/jbc.M103608200. 11375993

[glia70005-bib-0002] Anthony, D. C. , B. Ferguson , M. K. Matyzak , K. M. Miller , M. M. Esiri , and V. H. Perry . 1997. “Differential Matrix Metalloproteinase Expression in Cases of Multiple Sclerosis and Stroke.” Neuropathology and Applied Neurobiology 23, no. 5: 406–415. 10.1111/j.1365-2990.1997.tb01315.x. 9364466

[glia70005-bib-0003] Back, S. A. , T. M. F. Tuohy , H. Chen , et al. 2005. “Hyaluronan Accumulates in Demyelinated Lesions and Inhibits Oligodendrocyte Progenitor Maturation.” Nature Medicine 11, no. 9: 966–972. 10.1038/nm1279. 16086023

[glia70005-bib-0004] Bankhead, P. , M. B. Loughrey , J. A. Fernández , et al. 2017. “QuPath: Open Source Software for Digital Pathology Image Analysis.” Scientific Reports 7, no. 1: e16878. 10.1038/s41598-017-17204-5. PMC5715110 29203879

[glia70005-bib-0005] Baragi, V. M. , C. J. Fliszar , M. C. Conroy , Q. Z. Ye , J. M. Shipley , and H. G. Welgus . 1994. “Contribution of the C‐Terminal Domain of Metalloproteinases to Binding by Tissue Inhibitor of Metalloproteinases. C‐Terminal Truncated Stromelysin and Matrilysin Exhibit Equally Compromised Binding Affinities as Compared to Full‐Length Stromelysin.” Journal of Biological Chemistry 269, no. 17: 12692–12697. 8175679

[glia70005-bib-0006] Bredow, D. C. , R. B. Nagle , G. T. Bowden , and A. E. Cress . 1995. “Degradation of Fibronectin Fibrils by Matrilysin and Characterization of the Degradation Products.” Experimental Cell Research 221: 83–91. 7589259 10.1006/excr.1995.1355

[glia70005-bib-0007] Buhler, L. A. , R. Samara , E. Guzman , et al. 2009. “Matrix Metalloproteinase‐7 Facilitates Immune Access to the CNS in Experimental Autoimmune Encephalomyelitis.” BMC Neuroscience 10: 17. 10.1186/1471-2202-10-17. 19267908 PMC2660336

[glia70005-bib-0008] Chandler, S. , R. Coates , A. Gearing , J. Lury , G. Wells , and E. Bone . 1995. “Matrix Metalloproteinases Degrade Myelin Basic Protein.” Neuroscience Letters 201, no. 3: 223–226. 8786845 10.1016/0304-3940(95)12173-0

[glia70005-bib-0009] Chang, A. , W. W. Tourtellotte , R. Rudick , and B. D. Trapp . 2002. “Premyelinating Oligodendrocytes in Chronic Lesions of Multiple Sclerosis.” New England Journal of Medicine 346, no. 3: 165–173. 10.1056/NEJMoa010994. 11796850

[glia70005-bib-0010] Chari, D. M. , C. Zhao , M. R. Kotter , W. F. Blakemore , and R. J. M. Franklin . 2006. “Corticosteroids Delay Remyelination of Experimental Demyelination in the Rodent Central Nervous System.” Journal of Neuroscience Research 83, no. 4: 594–605. 10.1002/jnr.20763. 16429447

[glia70005-bib-0011] Chiao, Y. A. , R. Zamilpa , E. F. Lopez , et al. 2010. “In Vivo Matrix Metalloproteinase‐7 Substrates Identified in the Left Ventricle Post‐Myocardial Infarction Using Proteomics.” Journal of Proteome Research 9, no. 5: 2649–2657. 10.1021/pr100147r. 20232908 PMC2866015

[glia70005-bib-0012] Chung, C. Y. , and H. P. Erickson . 1997. “Glycosaminoglycans Modulate Fibronectin Matrix Assembly and Are Essential for Matrix Incorporation of Tenascin‐C.” Journal of Cell Science 110, no. 12: 1413–1419. 10.1242/jcs.110.12.1413. 9217327

[glia70005-bib-0013] Cooper, J. G. , S. J. Jeong , T. L. McGuire , et al. 2018. “Fibronectin EDA Forms the Chronic Fibrotic Scar After Contusive Spinal Cord Injury.” Neurobiology of Disease 116: 60–68. 10.1016/j.nbd.2018.04.014. 29705186 PMC5995671

[glia70005-bib-0014] Cossins, J. A. , J. M. Clements , J. Ford , et al. 1997. “Enhanced Expression of MMP‐7 and MMP‐9 in Demyelinating Multiple Sclerosis Lesions.” Acta Neuropathologica 94, no. 6: 590–598. 10.1007/s004010050754. 9444361

[glia70005-bib-0015] de Jong, J. M. , P. Wang , M. Oomkens , and W. Baron . 2020. “Remodeling of the Interstitial Extracellular Matrix in White Matter Multiple Sclerosis Lesions: Implications for Remyelination (Failure).” Journal of Neuroscience Research 98, no. 7: 1370–1397. 10.1002/jnr.24582. 31965607

[glia70005-bib-0016] Djuric, T. , and M. Zivkovic . 2017. “Overview of MMP Biology and Gene Associations in Human Diseases.” In The Role of Matrix Metalloproteinase in Human Body Pathologies. InTech. 10.5772/intechopen.70265.

[glia70005-bib-0017] Espitia Pinzon, N. , B. Sanz‐Morello , J. J. P. Brevé , et al. 2017. “Astrocyte‐Derived Tissue Transglutaminase Affects Fibronectin Deposition, but Not Aggregation, During Cuprizone‐Induced Demyelination.” Scientific Reports 7, no. 1: e40995. 10.1038/srep40995. PMC5269585 28128219

[glia70005-bib-0018] Fard, M. K. , F. Van der Meer , P. Sánchez , et al. 2017. “BCAS1 Expression Defines a Population of Early Myelinating Oligodendrocytes in Multiple Sclerosis Lesions.” Science Translational Medicine 9, no. 419: 1–28. 10.1126/scitranslmed.aam7816. PMC7116798 29212715

[glia70005-bib-0019] Franklin, R. J. M. , and M. Simons . 2022. “CNS Remyelination and Inflammation: From Basic Mechanisms to Therapeutic Opportunities.” Neuron 110, no. 21: 3549–3565. 10.1016/j.neuron.2022.09.023. 36228613

[glia70005-bib-0020] Gearing, A. J. H. , P. Beckett , M. Christodoulou , et al. 1994. “Processing of Tumour Necrosis Factor‐α Precursor by Metalloproteinases.” Nature 370, no. 6490: 555–557. 10.1038/370555a0. 8052310

[glia70005-bib-0021] Ghorbani, S. , and V. W. Yong . 2021. “The Extracellular Matrix as Modifier of Neuroinflammation and Remyelination in Multiple Sclerosis.” Brain 144, no. 7: 1958–1973. 10.1093/brain/awab059. 33889940 PMC8370400

[glia70005-bib-0022] Gorter, R. P. , and W. Baron . 2020. “Matrix Metalloproteinases Shape the Oligodendrocyte (Niche) During Development and Upon Demyelination.” Neuroscience Letters 729: e134980. 10.1016/j.neulet.2020.134980. 32315713

[glia70005-bib-0023] Greenfield, E. A. , J. Reddy , A. Lees , et al. 2006. “Monoclonal Antibodies to Distinct Regions of Human Myelin Proteolipid Protein Simultaneously Recognize Central Nervous System Myelin and Neurons of Many Vertebrate Species.” Journal of Neuroscience Research 83, no. 3: 415–431. 10.1002/jnr.20748. 16416423

[glia70005-bib-0024] Harauz, G. , and J. M. Boggs . 2013. “Myelin Management by the 18.5‐kDa and 21.5‐kDa Classic Myelin Basic Protein Isoforms.” Journal of Neurochemistry 125, no. 3: 334–361. 10.1111/jnc.12195. 23398367 PMC3700880

[glia70005-bib-0025] Haro, H. , H. C. Crawford , B. Fingleton , K. Shinomiya , D. M. Spengler , and L. M. Matrisian . 2000. “Matrix Metalloproteinase‐7–Dependent Release of Tumor Necrosis Factor‐α in a Model of Herniated Disc Resorption.” Journal of Clinical Investigation 105, no. 2: 143–150. 10.1172/JCI7091. 10642592 PMC377426

[glia70005-bib-0026] Hartenstein, B. , B. T. Dittrich , D. Stickens , et al. 2006. “Epidermal Development and Wound Healing in Matrix Metalloproteinase 13‐Deficient Mice.” Journal of Investigative Dermatology 126, no. 2: 486–496. 10.1038/sj.jid.5700084. 16374453 PMC2767339

[glia70005-bib-0027] Hill, K. E. , B. M. Lovett , and J. E. Schwarzbauer . 2022. “Heparan Sulfate Is Necessary for the Early Formation of Nascent Fibronectin and Collagen I Fibrils at Matrix Assembly Sites.” Journal of Biological Chemistry 298, no. 1: e101479. 10.1016/j.jbc.2021.101479. PMC8801470 34890641

[glia70005-bib-0028] Imai, K. , Y. Yokohamal , I. Nakanishi , et al. 1995. “Matrix Metalloproteinase 7 (Matrilysin) From Human Rectal Carcinoma Cells: Activation of the Precursor, Interaction With Other Matrix Metalloproteinases and Enzymic Properties.” Journal of Biological Chemistry 270, no. 12: 91–6697. 10.1074/jbc.270.12.6691. 7896811

[glia70005-bib-0029] Ineichen, B. V. , O. Weinmann , N. Good , et al. 2017. “Sudan Black: A Fast, Easy and Non‐Toxic Method to Assess Myelin Repair in Demyelinating Diseases.” Neuropathology and Applied Neurobiology 43, no. 3: 242–251. 10.1111/nan.12373. 28009439

[glia70005-bib-0030] Itoh, Y. 2015. “Membrane‐Type Matrix Metalloproteinases: Their Functions and Regulations.” Matrix Biology 44: 207–223. 10.1016/j.matbio.2015.03.004. 25794647

[glia70005-bib-0031] Kaiser, T. , H. M. Allen , O. Kwon , et al. 2021. “Myeltracer: A Semi‐Automated Software for Myelin G‐Ratio Quantification.” eNeuro 8, no. 4: 1–9. 10.1523/ENEURO.0558-20.2021. PMC8298095 34193510

[glia70005-bib-0032] Konstandin, M. H. , H. Toko , G. M. Gastelum , et al. 2013. “Fibronectin Is Essential for Reparative Cardiac Progenitor Cell Response After Myocardial Infarction.” Circulation Research 113, no. 2: 115–125. 10.1161/CIRCRESAHA.113.301152. 23652800 PMC3815660

[glia70005-bib-0033] Kuhlmann, T. , M. Moccia , T. Coetzee , et al. 2023. “Multiple Sclerosis Progression: Time for a New Mechanism‐Driven Framework.” Lancet Neurology 22, no. 1: 78–88. 10.1016/S1474-4422(22)00289-7. 36410373 PMC10463558

[glia70005-bib-0034] Kuipers, J. , and B. N. G. Giepmans . 2020. “Neodymium as an Alternative Contrast for Uranium in Electron Microscopy.” Histochemistry and Cell Biology 153, no. 4: 271–277. 10.1007/s00418-020-01846-0. 32008069 PMC7160090

[glia70005-bib-0035] Lassmann, H. 2018. “Multiple Sclerosis Pathology.” Cold Spring Harbor Perspectives in Medicine 8, no. 3: a028936. 10.1101/cshperspect.a028936. 29358320 PMC5830904

[glia70005-bib-0036] Li, Q. , P. W. Park , C. L. Wilson , and W. C. Parks . 2002. “Matrilysin Shedding of Syndecan‐1 Regulates Chemokine Mobilization and Transepithelial Efflux of Neutrophils in Acute Lung Injury.” Cell 111, no. 5: 635–646. 10.1016/s0092-8674(02)01079-6. 12464176

[glia70005-bib-0037] Liu, X. , H. Wu , M. Byrne , J. Jeffrey , S. Krane , and R. Jaenisch . 1995. “A Targeted Mutation at the Known Collagenase Cleavage Site in Mouse Type I Collagen Impairs Tissue Remodeling.” Journal of Cell Biology 130, no. 1: 227–237. 7790374 10.1083/jcb.130.1.227 PMC2120510

[glia70005-bib-0038] Lucchinetti, C. , W. Brück , J. Parisi , B. Scheithauer , M. Rodriguez , and H. Lassmann . 1999. “A Quantitative Analysis of Oligodendrocytes in Multiple Sclerosis Lesions A Study of 113 Cases.” Brain 122: 2279–2295. 10.1093/brain/122.12.2279. 10581222

[glia70005-bib-0039] Luchetti, S. , N. L. Fransen , C. G. van Eden , V. Ramaglia , M. Mason , and I. Huitinga . 2018. “Progressive Multiple Sclerosis Patients Show Substantial Lesion Activity That Correlates With Clinical Disease Severity and Sex: A Retrospective Autopsy Cohort Analysis.” Acta Neuropathologica 135, no. 4: 511–528. 10.1007/s00401-018-1818-y. 29441412 PMC5978927

[glia70005-bib-0040] Mao, Y. , and J. E. Schwarzbauer . 2005. “Fibronectin Fibrillogenesis, a Cell‐Mediated Matrix Assembly Process.” Matrix Biology 24, no. 6: 389–399. 10.1016/j.matbio.2005.06.008. 16061370

[glia70005-bib-0041] Milward, E. , K. J. Kim , A. Szklarczyk , et al. 2008. “Cleavage of Myelin Associated Glycoprotein by Matrix Metalloproteinases.” Journal of Neuroimmunology 193, no. 1–2: 140–148. 10.1016/j.jneuroim.2007.11.001. 18063113 PMC2276728

[glia70005-bib-0042] Nguyen, T. T. , W. R. Wolter , B. Anderson , et al. 2020. “Limitations of Knockout Mice and Other Tools in Assessment of the Involvement of Matrix Metalloproteinases in Wound Healing and the Means to Overcome Them.” ACS Pharmacology & Translational Science 3, no. 3: 489–495. 10.1021/acsptsci.9b00109. 32566914 PMC7296540

[glia70005-bib-0043] Oost, W. , A. J. Huitema , K. Kats , et al. 2023. “Pathological Ultrastructural Alterations of Myelinated Axons in Normal Appearing White Matter in Progressive Multiple Sclerosis.” Acta Neuropathologica Communications 11, no. 1: e100. 10.1186/s40478-023-01598-7. PMC10283269 37340488

[glia70005-bib-0044] Page‐McCaw, A. , A. J. Ewald , and Z. Werb . 2007. “Matrix Metalloproteinases and the Regulation of Tissue Remodelling.” Nature Reviews. Molecular Cell Biology 8, no. 3: 221–233. 10.1038/nrm2125. 17318226 PMC2760082

[glia70005-bib-0045] Paxinos, G. , and K. B. J. Franklin . 2001. The Mouse Brain in Stereotactic Coordinates. 2nd ed. Academic Press.

[glia70005-bib-0046] Qin, J. , A. H. Sikkema , K. van der Bij , et al. 2017. “GD1a Overcomes Inhibition of Myelination by Fibronectin via Activation of Protein Kinase A: Implications for Multiple Sclerosis.” Journal of Neuroscience 37, no. 41: 9925–9938. 10.1523/JNEUROSCI.0103-17.2017. 28899916 PMC6596593

[glia70005-bib-0047] Rahimi, Z. , Z. Abdan , Z. Rahimi , et al. 2016. “Functional Ppromoter Polymorphisms of MMP‐2 C‐735T and MMP‐9 C‐1562T and Their Synergism With MMP‐7 A‐181G in Multiple Sclerosis.” Immunological Investigations 45, no. 6: 543–552. 10.1080/08820139.2016.1180303. 27409770

[glia70005-bib-0048] Rahimi, Z. , Z. Rahimi , F. Mohammadi , N. Razazian , and F. Najafi . 2014. “Association of Matrix Metalloproteinase‐7A‐181G Variants With the Risk of Multiple Sclerosis.” Personalized Medicine 11, no. 8: 727–733. 10.2217/pme.14.42. 29764043

[glia70005-bib-0049] Raitman, I. , M. L. Huang , S. A. Williams , B. Friedman , K. Godula , and J. E. Schwarzbauer . 2018. “Heparin‐Fibronectin Interactions in the Development of Extracellular Matrix Insolubility.” Matrix Biology 67: 107–122. 10.1016/j.matbio.2017.11.012. 29223498 PMC5910196

[glia70005-bib-0050] Rodrigues, A. , C. Nogueira , L. C. Marinho , et al. 2022. “Computer‐Assisted Tumor Grading, Validation of PD‐L1 Scoring, and Quantification of CD8‐Positive Immune Cell Density in Urothelial Carcinoma, a Visual Guide for Pathologists Using QuPath.” Surgical and Experimental Pathology 5, no. 1: e12. 10.1186/s42047-022-00112-y.

[glia70005-bib-0051] Rodríguez, D. , C. J. Morrison , and C. M. Overall . 2010. “Matrix Metalloproteinases: What Do They Not Do? New Substrates and Biological Roles Identified by Murine Models and Proteomics.” Biochimica et Biophysica Acta 1803, no. 1: 39–54. 10.1016/J.BBAMCR.2009.09.015. 19800373

[glia70005-bib-0052] Schindelin, J. , I. Arganda‐Carreras , E. Frise , et al. 2012. “Fiji: An Open‐Source Platform for Biological‐Image Analysis.” Nature Methods 9, no. 7: 676–682. 10.1038/nmeth.2019. 22743772 PMC3855844

[glia70005-bib-0053] Sherman, L. S. , S. Matsumoto , W. Su , T. Srivastava , and S. A. Back . 2015. “Hyaluronan Synthesis, Catabolism, and Signaling in Neurodegenerative Diseases.” International Journal of Cell Biology 2015: 1–10. 10.1155/2015/368584. PMC4581574 26448752

[glia70005-bib-0054] Siri, A. , V. Knauper , N. Veirana , F. Caocci , G. Murphy , and L. Zardi . 1995. “Different Susceptibility of Small and Large Human Tenascin‐C Isoforms to Degradation by Matrix Metalloproteinases.” Journal of Biological Chemistry 270, no. 15: 8650–8654. 10.1074/jbc.270.15.8650. 7536739

[glia70005-bib-0055] Skuljec, J. , V. Gudi , R. Ulrich , et al. 2011. “Matrix Metalloproteinases and Their Tissue Inhibitors in Cuprizone‐Induced Demyelination and Remyelination of Brain White and Gray Matter.” Journal of Neuropathology and Experimental Neurology 70, no. 9: 758–769. 10.1097/NEN.0b013e3182294fad. 21865884

[glia70005-bib-0056] Sloane, J. A. , C. Batt , Y. Ma , Z. M. Harris , B. Trapp , and T. Vartanian . 2010. “Hyaluronan Blocks Oligodendrocyte Progenitor Maturation and Remyelination Through TLR2.” Proceedings of the National Academy of Sciences of the United States of America 107, no. 25: 11555–11560. 10.1073/pnas.1006496107. 20534434 PMC2895128

[glia70005-bib-0057] Sobel, R. A. , and M. E. Mitchell . 1989. “Fibronectin in Multiple Sclerosis Lesions.” American Journal of Pathology 135, no. 1: 161–168. 2528301 PMC1880224

[glia70005-bib-0058] Stoffels, J. M. J. , J. C. de Jonge , M. Stancic , et al. 2013. “Fibronectin Aggregation in Multiple Sclerosis Lesions Impairs Remyelination.” Brain 136, no. 1: 116–131. 10.1093/brain/aws313. 23365094

[glia70005-bib-0059] Stoffels, J. M. J. , D. Hoekstra , R. J. M. Franklin , W. Baron , and C. Zhao . 2015. “The EIIIA Domain From Astrocyte‐Derived Fibronectin Mediates Proliferation of Oligodendrocyte Progenitor Cells Following CNS Demyelination.” Glia 63: 242–256. 10.1002/glia.22748. 25156142 PMC4737254

[glia70005-bib-0060] Stoffels, J. M. J. , C. Zhao , and W. Baron . 2013. “Fibronectin in Tissue Regeneration: Timely Disassembly of the Scaffold Is Necessary to Complete the Build.” Cellular and Molecular Life Sciences 70, no. 22: 4243–4253. 10.1007/s00018-013-1350-0. 23756580 PMC11113129

[glia70005-bib-0061] Tammi, R. H. , A. G. Passi , K. Rilla , et al. 2011. “Transcriptional and Post‐Translational Regulation of Hyaluronan Synthesis.” FEBS Journal 278, no. 9: 1419–1428. 10.1111/j.1742-4658.2011.08070.x. 21362137

[glia70005-bib-0062] Trapp, B. D. , and K. A. Nave . 2008. “Multiple Sclerosis: An Immune or Neurodegenerative Disorder?” Annual Review of Neuroscience 31: 247–269. 10.1146/annurev.neuro.30.051606.094313. 18558855

[glia70005-bib-0063] van Horssen, J. , L. Bö , C. M. P. Vos , I. Virtanen , and H. E. de Vries . 2005. “Basement Membrane Proteins in Multiple Sclerosis‐Associated Inflammatory Cuffs: Potential Role in Influx and Transport of Leukocytes.” Journal of Neuropathology and Experimental Neurology 64, no. 8: 722–729. 10.1097/01.jnen.0000173894.09553.13. 16106221

[glia70005-bib-0064] Vega‐Riquer, J. M. , G. Mendez‐Victoriano , R. A. Morales‐Luckie , and O. Gonzalez‐Perez . 2019. “Five Decades of Cuprizone, an Updated Model to Replicate Demyelinating Diseases.” Current Neuropharmacology 17, no. 2: 129–141. 10.2174/1570159x15666170717120343. 28714395 PMC6343207

[glia70005-bib-0065] Wang, P. , R. P. Gorter , J. C. de Jonge , et al. 2018. “MMP7 Cleaves Remyelination‐Impairing Fibronectin Aggregates and Its Expression Is Reduced in Chronic Multiple Sclerosis Lesions.” Glia 66, no. 8: 1625–1643. 10.1002/glia.23328. 29600597 PMC6099312

[glia70005-bib-0066] Werkman, I. , A. H. Sikkema , J. B. Versluijs , J. Qin , P. de Boer , and W. Baron . 2020. “TLR3 Agonists Induce Fibronectin Aggregation by Activated Astrocytes: A Role of Pro‐Inflammatory Cytokines and Fibronectin Splice Variants.” Scientific Reports 10, no. 1: e532. 10.1038/s41598-019-57069-4. PMC6969115 31953424

[glia70005-bib-0067] Wierzbicka‐Patynowski, I. , Y. Mao , and J. E. Schwarzbauer . 2004. “Analysis of Fibronectin Matrix Assembly.” In Current Protocols in Cell Biology, vol. 10. Princeton University. 10.1002/0471143030.cb1012s25 18228438

[glia70005-bib-0068] Wilson, C. L. , K. J. Heppner , P. A. Labosky , B. L. Hogan , L. M. Matrisian , and R. Sager . 1997. “Intestinal Tumorigenesis Is Suppressed in Mice Lacking the Metalloproteinase Matrilysin.” Medical Science 94: 1402–1407. 10.1073/pnas.94.4.1402. PMC19803 9037065

[glia70005-bib-0069] Wu, N. , E. D. Jansen , and J. M. Davidson . 2003. “Comparison of Mouse Matrix Metalloproteinase 13 Expression in Free‐Electron Laser and Scalpel Incisions During Wound Healing.” Journal of Investigative Dermatology 121, no. 4: 926–932. 10.1046/j.1523-1747.2003.12497.x. 14632214

[glia70005-bib-0070] Zack, M. D. , A. M. Malfait , A. P. Skepner , et al. 2009. “ADAM‐8 Isolated From Human Osteoarthritic Chondrocytes Cleaves Fibronectin at Ala271.” Arthritis and Rheumatism 60, no. 9: 2704–2713. 10.1002/art.24753. 19714641

[glia70005-bib-0071] Žádníková, P. , R. Šínová , V. Pavlík , et al. 2022. “The Degradation of Hyaluronan in the Skin.” Biomolecules 12, no. 2: e251. 10.3390/biom12020251. PMC8961566 35204753

[glia70005-bib-0072] Zeidán‐Chuliá, F. , D. Yilmaz , L. Häkkinen , et al. 2018. “Matrix Metalloproteinase‐7 in Periodontitis With Type 2 Diabetes Mellitus.” Journal of Periodontal Research 53, no. 5: 916–923. 10.1111/jre.12583. 29974476

[glia70005-bib-0073] Zhan, J. , T. Mann , S. Joost , N. Behrangi , M. Frank , and M. Kipp . 2020. “The Cuprizone Model: Dos and Do Nots.” Cells 9, no. 4: e843. 10.3390/CELLS9040843. PMC7226799 32244377

[glia70005-bib-0074] Zhou, D. , Y. Tian , L. Sun , et al. 2017. “Matrix Metalloproteinase‐7 Is a Urinary Biomarker and Pathogenic Mediator of Kidney Fibrosis.” Journal of the American Society of Nephrology 28, no. 2: 598–611. 10.1681/ASN.2016030354. 27624489 PMC5280025

