# Supplementary material for: Unexpected Remyelination in the Absence of Matrix Metalloproteinase 7
Source: Glia. 2025 Mar 10;73(6):1288–306. doi: 10.1002/glia.70005 (PMC12012326; doi:10.1002/glia.70005)
Supplement: Supplementary file 1 — Figure S1. [file GLIA-73-1288-s001.pdf]

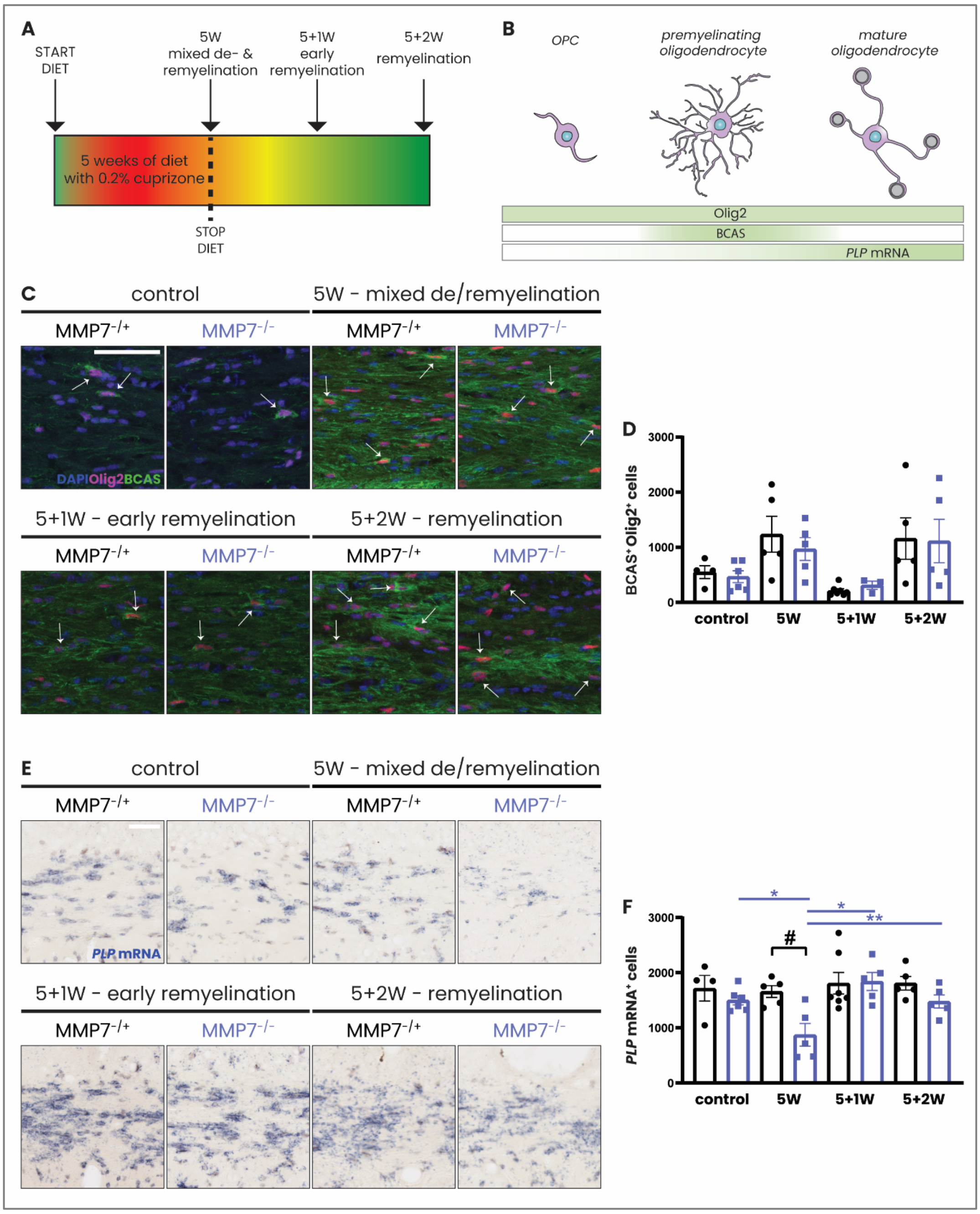

SUPPLEMENTARY FIGURE 1: Absence of MMP7 subtly impairs the temporal dynamics of OPC differentiation during remyelination. **(A)** Experimental set-up: eight-week-old male and female MMP7$^{-/+}$ and MMP7$^{-/-}$ mice were fed with regular chow (control), with 0.2% cuprizone-containing chow for five weeks (mixed demyelination/remyelination) or with 0.2% cuprizone for five weeks followed by recovery on a normal diet for one week (early remyelination) or two weeks (remyelination). Across all cuprizone experiments, the same group of thirteen-week-old MMP7$^{-/+}$ or MMP7$^{-/-}$ mice fed with regular chow served as controls. **(B)** Schematic representation of the expression of Olig2 (oligodendrocyte lineage marker), BCAS1 (pre-myelinating oligodendrocyte marker) and *PLP* mRNA (marker transcriptionally upregulated by actively myelinating oligodendrocytes) during oligodendrocyte maturation. **(C)** Representative images of immunofluorescent staining of the corpus callosum for Olig2 (pink), and BCAS1 (green). Nuclei were visualised with DAPI (blue). BCAS1^{+} oligodendrocytes are highlighted with white arrows. **(D)** Quantification of BCAS1^{+}Olig2^{+} cells in the corpus callosum. **(E)** Representative images of *in-situ* hybridization of the corpus callosum for *PLP* mRNA (blue). **(F)** Quantification of PLP mRNA^{+} cells in the corpus callosum. Data are presented as the mean ± the SEM. MMP7$^{-/+}$ mice are represented as black dots and MMP7$^{-/-}$ mice as blue squares. To assess changes between different timepoints, statistical analyses were performed using one-way ANOVA followed by Tukey's multiple comparisons test. In case of unequal variance, Welch's ANOVA test followed by Dunnet's T3 multiple comparisons test was used. To assess changes between MMP7$^{-/+}$ and MMP7$^{-/-}$ mice, unpaired T-tests or, in case of unequal variance, unpaired T-tests with Welch's correction were used. Significant data between timepoints (*$p < 0.05$, **$p < 0.01$) and between bMMP7$^{-/+}$ and MMP7$^{-/-}$ mice ($^{\#}p < 0.05$) are presented. Scale bars are 50µm.
